# Loss of HIF-1α in natural killer cells inhibits tumour growth by stimulating non-productive angiogenesis

**DOI:** 10.1038/s41467-017-01599-w

**Published:** 2017-11-17

**Authors:** Ewelina Krzywinska, Chahrazade Kantari-Mimoun, Yann Kerdiles, Michal Sobecki, Takayuki Isagawa, Dagmar Gotthardt, Magali Castells, Johannes Haubold, Corinne Millien, Thomas Viel, Bertrand Tavitian, Norihiko Takeda, Joachim Fandrey, Eric Vivier, Veronika Sexl, Christian Stockmann

**Affiliations:** 1 Institut National de la Santé et de la Recherche Médicale (INSERM), Paris Cardiovascular Research Center, Unit 970, 56 Rue Leblanc, Paris, 75015 France; 2 Centre d’Immunologie de Marseille-Luminy, Aix Marseille Université UM2, Inserm, U1104, CNRS UMR7280, Avenue de Luminy, Marseille, 13288 France; 3 Institut de Biologie Intégrative de la Cellule (I2BC), Genome Biology, CEA/CNRS/Université Paris Sud, Avenue de la Terrasse, Gif sur Yvette, Paris, 91190 France; 4 Graduate School of Biomedical Science, Nagasaki University, 1-12-4 Sakamoto, Nagasaki, 852-8523 Japan; 5 Institute of Pharmacology and Toxicology, University of Veterinary Medicine Vienna, Veterinärplatz 1, Viena, 1210 Austria; 6 Institut für Physiologie, Universitätsklinikum Essen, Universität Duisburg-Essen, Essen, 45147 Germany; 7 Department of Cardiovascular Medicine, Graduate School of Medicine, The University of Tokyo, Tokyo, 113-8655 Japan; 8 Immunologie, Hôpital de la Conception, Assistance Publique—Hôpitaux de Marseille, Aix-Marseille Université, Marseille, 13005 France; 9 Institute of Anatomy, University of Zurich, Winterthurerstrasse 190, Zurich, CH-8057 Switzerland

## Abstract

Productive angiogenesis, a prerequisite for tumour growth, depends on the balanced release of angiogenic and angiostatic factors by different cell types within hypoxic tumours. Natural killer (NK) cells kill cancer cells and infiltrate hypoxic tumour areas. Cellular adaptation to low oxygen is mediated by Hypoxia-inducible factors (HIFs). We found that deletion of HIF-1α in NK cells inhibited tumour growth despite impaired tumour cell killing. Tumours developing in these conditions were characterised by a high-density network of immature vessels, severe haemorrhage, increased hypoxia, and facilitated metastasis due to non-productive angiogenesis. Loss of HIF-1α in NK cells increased the bioavailability of the major angiogenic cytokine vascular endothelial growth factor (VEGF) by decreasing the infiltration of NK cells that express angiostatic soluble VEGFR-1. In summary, this identifies the hypoxic response in NK cells as an inhibitor of VEGF-driven angiogenesis, yet, this promotes tumour growth by allowing the formation of functionally improved vessels.

## Abstract (web summary)

Tumour hypoxia influences both the immune responses and angiogenesis. Here, the authors show that HIF-1α deletion in NK cells impairs NK cytotoxic activity but inhibit tumour growth by decreasing the infiltration of NK cells that express angiostatic soluble VEGFR-1, thus resulting in non-functional angiogenesis.

## Introduction

Angiogenesis is required for tumour progression, and involves release of angiogenic factors, including vascular endothelial growth factor (VEGF)^1,2^. In most tumours, despite high vascular density, the vasculature differs from normal vascular networks and is characterised by an inefficient blood supply. Vessel abnormalities include increased permeability and haemorrhage as well as decreased pericyte coverage, which frequently cause tumour hypoxia and increased metastasis^3^. Therefore, angiostatic factors that counteract VEGF signalling are also required for the formation of functional blood vessels and the prevention of excessive angiogenesis^3–5^. Hence, productive angiogenesis depends on the balanced release of angiogenic and angiostatic factors from both malignant and stromal cell types^3–7^.

Natural killer (NK) cells are a subset of cytotoxic innate lymphoid cells with a unique capacity to kill cancer cells and restrict tumour growth as well as metastatic spread^8^. Therefore, adoptive NK cell transfer becomes increasingly important for the treatment of various types of cancer^8^. Moreover, NK cells are believed to contribute to physiological angiogenesis during pregnancy via the release of angiogenic factors^9^. Yet, the role of NK cells in pathological tumour angiogenesis remains ill defined. Tumour infiltrating NK cells are likely required to operate in hypoxic conditions and cellular adaptation to low oxygen is mediated by Hypoxia-inducible transcription factors (HIFs), with HIF-1 and HIF-2 being the most extensively studied^10–12^. It is commonly accepted that the hypoxic response plays a pivotal role in guiding immune responses as well as driving angiogenesis^12,13^. Noteworthy, whereas adaptive immune responses may be impaired by low oxygen, innate immune cells show a pro-proangiogenic and proinflammatory response during hypoxia and HIF-1 activation^12,13^. Since NK cells unify features of both, innate as well as adaptive immunity, it was key to study the impact of the hypoxic response in this cell type.

## Results

### HIF-1α depletion impairs NK cell function and tumour growth

**Fig. 1:**
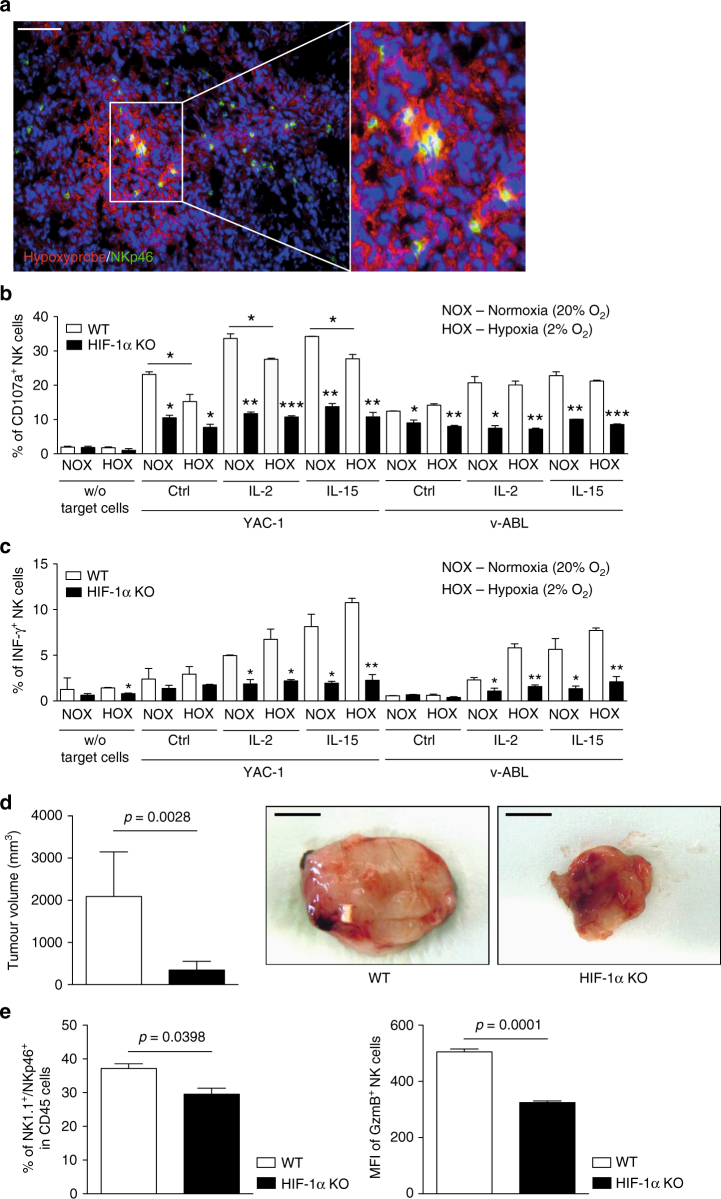
HIF-1α depletion in NK cells results in decreased cytotoxicity but delays tumour growth. **a** Representative image of tumour hypoxia in LLC isografts with the specific markers Hypoxyprobe (red), NKp46 (green), and nuclei (blue). **b** Splenocytes from WT and HIF-1α KO mice were stimulated with target cells (YAC-1 and V-abl lymphoma cells, Ratio E/T 1:1), in absence or presence of rhIL-2 and rmIL15. NK cell degranulation (CD107A^+^) and **c** INF-γ expression were analysed by flow cytometry (*n* = 4 for each group). **d** Tumour volume analysis of V-abl tumours injected subcutaneously in WT and HIF-1α KO mice with representative images at endpoint, day 21 (*n* = 11 for each group). Scale bars in macroscopic figures indicate 5 mm. **e** Flow cytometry analysis for NK1.1, NKp46 (for NK cells), GzmB (for NK cell activation state) on V-abl tumours from WT and HIF-1α KO mice at endpoint, day 21 (*n* = 3). Statistical significance was determined by an unpaired Student’s *t*-test or one-way analysis of variance, where appropriate. Bars represent mean values; error bars indicate the s.e.m. Statistical significance is indicated as *P < 0.05, **P < 0.01, and ***P < 0.001. Scale bar, 100 μm

Prompted by the observation that NKp46-expressing NK cells infiltrate hypoxic tumours (Fig. 1a), and in order to test the role of HIF-1α in NK cells, we created an in vivo, targeted deletion of HIF-1α in NK cells, via crosses of the loxP-flanked HIF-1α allele^14^ to the *Ncr1* (NKp46) promoter-driven Cre recombinase^15,16^, specific to NKp46-expressing innate lymphoid cells^17^, including NK cells (*HIF-1α* ^*fl+/fl+/*^ *Ncr1cre + *mice, termed HIF-1α KO). This results in efficient deletion of HIF-1α at the mRNA and protein levels in isolated splenic NK cells (Supplementary Fig. 1a).

NK cell reactivity is strongly linked to NK cell maturation which can be distinguished by the expression of CD27/CD11b^18^ along with the development of a repertoire of inhibiting and activating receptors^19^. Importantly, despite reduced numbers of splenic NK cells, loss of HIF-1α did neither affect maturation, nor the receptor repertoire of NK cells from different organs (Supplementary Fig. 1b–e).

To extend earlier reports on hypoxia and NK cell effector function^20^, and to specifically determine the impact of HIF-1α on NK cell responsiveness, splenocytes from wild-type (WT) and HIF-1α KO mice were stimulated with ligand-mediated activation of the NK1.1 receptor, activating cytokines IL-12 and IL-18 or unspecific activation by PMA/ionomycin under normoxia (20% O_2_) and hypoxia (2% O_2_) for 6 h in vitro. In this setting, neither hypoxia nor loss of HIF-1α had any effect on NK cell reactivity as demonstrated by NK cell degranulation (CD107A+) and IFN-γ expression (Supplementary Fig. 1f). However, upon challenge with the tumour target cells YAC-1 and V-abl^21^, loss of HIF-1α reduced the fraction of CD107A-positive and IFN-γ-positive NK cells in normoxia and hypoxia (Fig. 1b, c). This hyporeactivity was not due to differences in surface receptor expression or viability across genotypes (Supplementary Fig. 1g, h) and could not be rescued by cytokine stimulation with IL-2 or IL-15 (Fig. 1b, c). These results indicated that HIF-1α is required for NK cell-mediated tumour cell killing.

In order to address the impact of HIF-1α on NK cell-dependent tumour growth control in vivo, we conducted experiments with subcutaneous isografts of V-abl lymphomas that are subject to NK cell-mediated killing^21^. Surprisingly, HIF-1α KO mice showed a reduction of tumour volumes of more than 80% compared to tumours from WT littermates at endpoint (day 18; Fig. 1d). Flow cytometry data analysis showed that infiltration of CD4 and CD8 lymphocytes was similar across genotypes (Supplementary Fig. 1i). Yet, despite reduced tumour burden in HIF-1α KO mice, the number of tumour infiltrating NK cells and as well as the expression of the cytotoxic effector molecule Granzyme B in NK cells was lower than in lymphomas from WT littermates (Fig. 1e). These data suggest that reduced lymphoma volumes in HIF-1α KO mice occur independently of NK cell-mediated tumour cell killing.

### HIF-1α in NK cells slows down angiogenesis

**Fig. 2:**
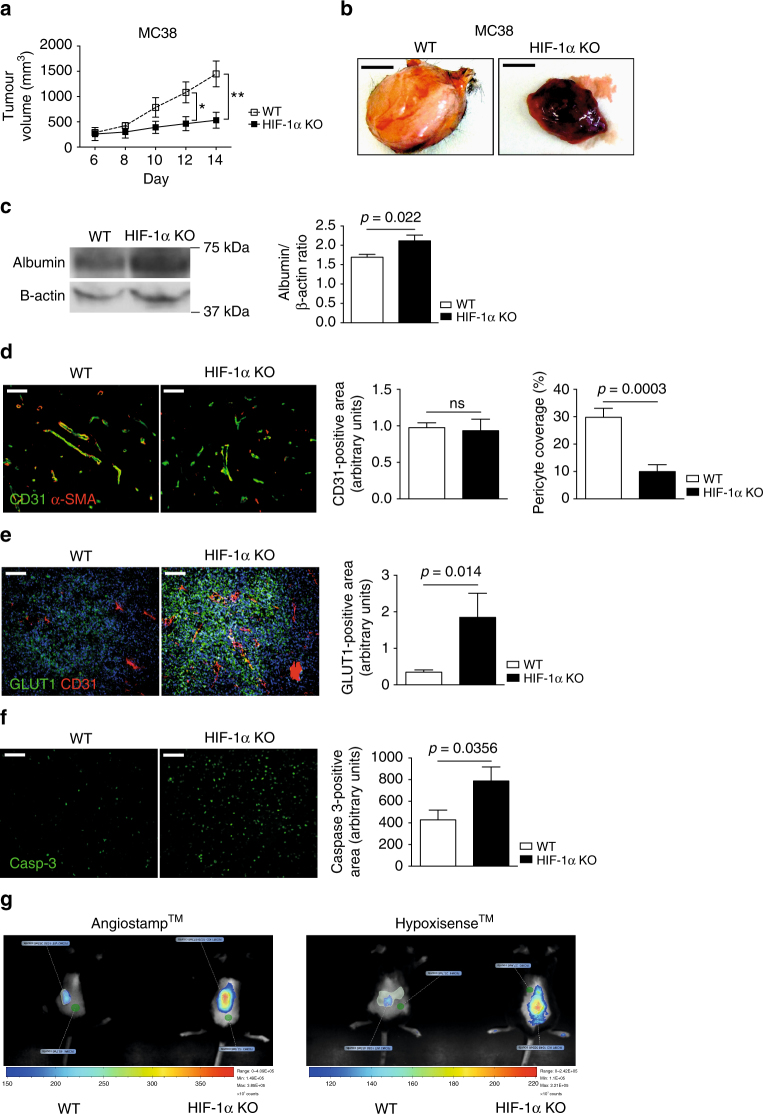
Loss of HIF-1α in NK cells impairs tumour growth and promotes non-functional angiogenesis. **a** Tumour volume analysis of MC38 isografts injected subcutaneously in WT and HIF-1α KO mice (*n* = 10 for each group), **b** and representative images at endpoint, day 14. Scale bars in macroscopic figures indicate 5 mm. **c** Left: Western blot of albumin and β-actin for MC38 tumours from WT and HIF-1α KO mice; Right: Quantitative analysis of intratumoural amounts of albumin for MC38 isografts from WT and HIF-1α KO mice (*n* = 8). **d** Left: representative images of simultaneous immunodetection of endothelial cells and pericytes in MC38 tumours with the specific markers CD31 and alpha smooth muscle actin (α-SMA); Right: Quantitative analysis of CD31-positive endothelial cells in WT and HIF-1α KO mice (*n* = 8) and pericyte coverage as assessed by α-SMA/CD31 co-localisation (*n* = 8). **e** Left: representative images of tumour hypoxia in MC38 isografts with the specific markers CD31 and GLUT1; Right: Quantitative analysis of hypoxic tumour areas (*n* = 8). **f** Left: representative images of caspase-3-positive area in MC38 tumours; Right: Quantitative analysis of caspase-3-positive areas (*n* = 10). **g** Representative images of non-invasive fluorescence molecular real-time imaging with the fluorescent probes AngioSense^®^ and HypoxiSense^®^ for in vivo monitoring of angiogenesis and tumour oxygenation at day 8 after tumour inocculation. Statistical significance was determined by an unpaired Student’s *t*-test or one-way analysis of variance, where appropriate. Bars represent mean values; error bars indicate the s.e.m. Statistical significance is indicated as *P < 0.05, **P < 0.01, and ***P < 0.001. Scale bar, 100 μm

To test this hypothesis, we analysed the effect of NK cell HIF-1α deficiency in tumours that are less susceptible to NK cell-mediated lysis as confirmed by in vitro activation and in vitro cytotoxicity assays (Supplementary Fig. 2a). Exposure of “less-susceptible” colon carcinoma (MC38) and Lewis Lung Carcinoma (LLC) cells to NK cells resulted in no activation and less than 2% of specific tumour cell lysis, regardless of the genotype or effector:target ratio (Supplementary Fig. 2a). In contrast, co-culture of “susceptible” V-abl cells with WT or HIF-1α KO NK cells, resulted in killing efficiencies up to 75 and 50%, respectively. To this end, we challenged our mice with subcutaneous MC38 and LLC cells isografts. Again, tumours in HIF-1α KO mice had significantly lower volumes at day 14 (Fig. 2a, b and Supplementary Fig. 2b), despite a reduction in NK cell infiltration and a lower fraction of degranulating CD107A^+^ NK cells (Supplementary Fig. 2c). No genotype-specific differences in infiltration of other immune cell subsets were detectable (Supplementary Fig. 2c), further indicating that impaired tumour growth in HIF-1α KO mice does not primarily rely on NK cell cytotoxicity. Interestingly, quantitative assessment of NK cell localisation relative to hypoxic areas within the tumour by means of double immunofluorescence for NKp46 and the hypoxia-inducible surrogate marker glucose transporter-1 (Glut1) revealed that HIF-1α KO NK cells preferentially accumulated in well oxygenated areas of the tumour and were less abundant in hypoxic zones (Supplementary Fig. 2d).

Macroscopic inspection of tumours revealed severe tumour haemorrhage in isografts from HIF-1α KO mice (Fig. 2b and Supplementary Fig. 2b), indicating an immature vascular phenotype with excessive leakage. Therefore, we quantified intratumoural amounts of albumin, a large serum protein that does not extravasate across an intact vascular barrier and therefore is indicative of pathologically increased vascular permeability. Consistently, tumours from HIF-1α KO mice showed an increase in extravasated albumin (Fig. 2c and Supplementary Fig. 2e). This prompted us to analyse the tumour vasculature in detail. Quantitative analysis of tumour vessel density and pericyte coverage was then carried out using CD31 and α-SMA, respectively, as markers for immunostaining. As shown in Fig. 2d and Supplementary Fig. 2f, loss of HIF-1α in NK cells resulted in a marked decrease of pericyte coverage, whereas overall tumour vessel density did not change. This suggests that the hypoxic response in tumour-infiltrating NK cells is essential to prevent pericyte loss and to preserve a more mature vessel phenotype during remodelling of the tumour vasculature.

Hypoxia is a typical feature of solid tumours and often a result of an immature and non-functional vasculature, despite the presence of a high-density vascular network^3,22^. We found that the more slowly growing tumours from HIF-1α KO mice had markedly increased levels of hypoxia determined by Glut1 staining (Fig. 2e and Supplementary Fig. 2g), which also occurred in areas with high vessel density along with increased tumour cell death as assessed by caspase 3 staining (Fig. 2f and Supplementary Fig. 2h). This striking uncoupling of angiogenesis and tumour oxygenation in HIF-1α KO mice was confirmed in an independent experimental setup. We co-injected the fluorescent probes AngioSense^®^, that binds to endothelial integrins that are exposed during vascular remodelling and HypoxiSense^®^, that binds to hypoxia-inducible carbonic anhydrase IX, into tumour-bearing mice at day 8 post-tumour inocculation when tumour volumes are still similar across genotypes (Fig. 2a). In vivo real-time imaging of both probes then allows monitoring of angiogenic activity and tumour hypoxia. This revealed increased tumour hypoxia despite increased angiogenic activity HIF-1α KO mice (Fig. 2g) that preceeds differences in tumour growth kinetics (Fig. 2a). This led us to conclude that loss of HIF-1α in NK cells induces non-productive angiogenesis.

### NK cell HIF-1α deficiency increases VEGF bioavailability

**Fig. 3:**
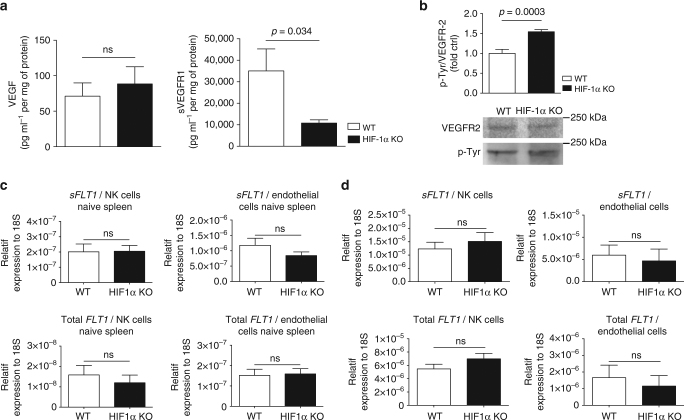
NK cell HIF-1α deficiency increases VEGF bioavailability and endothelial cell migration. **a** Determination of levels of VEGF and sVEGFR1 protein in MC38 isografts implanted in WT and HIF-1α KO mice by ELISA at endpoint, day 14 (*n* = 8). **b** Immunoblotting for VEGFR2 and phosphotyrosine (p-Tyr) after immunoprecipitation of VEGFR2 from MC38 tumour lysates and ratio of p-Tyr and VEGFR2 signal intensities as measured by photon emission at endpoint, day 14 (*n* = 8). **c** Gene expression analysis for soluble variant of *FLT1* and total form of *FLT1* on sorted NK cells and endothelial cells from naïve spleens from WT and HIF-1α KO mice (*n* = 6). **d** Gene expression analysis for soluble variant of *FLT1* and total form of *FLT1* on sorted intratumoural NK cells and endothelial cells from MC38 tumours injected subcutaneously in WT and HIF-1α KO mice at endpoint, day 10 (*n* = 10). Statistical significance was determined by an unpaired Student’s *t*-test or one-way analysis of variance, where appropriate. Bars represent mean values; error bars indicate the s.e.m. Statistical significance is indicated as *P < 0.05, **P < 0.01, and ***P < 0.001

Functional angiogenesis and the formation of mature vessels require the balanced release of angiogenic and angiostatic factors^3,4,6^. Gene expression analysis for angiogenic and angiostatic factors at endpoint (Supplementary Fig. 3a) revealed, in addition to changes in fibroblast growth factor-2 and angiopoietin-2 expression, a marked decrease in the expression of the angiostatic soluble form of VEGF receptor 1 (*sVEGFR1*) in tumours from HIF-1α KO mice, whereas *VEGF* expression was similar across genotypes (Supplementary Fig. 3a). This pattern was confirmed on tumour protein lysates by ELISA (Fig. 3a and Supplementary Fig. 3b). sVEGFR1 binds and sequesters VEGF with high affinity, thus reducing VEGF bioavailability and angiogenic signalling in the tumour microenvironment^4,23^. Hence, we determined whether VEGF-dependent signalling to the tumour endothelium was affected by the loss of HIF-1α in NK cells. VEGFR2 is an endothelial cell-specific receptor tyrosine kinase that is critical for VEGF signalling^23^. By immunoprecipitating VEGFR2 from tumour lysates and probing with anti-phosphotyrosine followed by anti-VEGFR2 antibody via Western blot, we quantified total and activated VEGFR2 from whole tumour lysates^6^. As shown in Fig. 3b and Supplementary Fig. 3c, loss of HIF-1α in NK cells significantly increased the ratio of phosphorylated VEGFR2 relative to total VEGFR2, when compared to WT conditions. The reduction in sVEGFR1 levels and subsequently enhanced VEGFR2 activation suggests that NK cells critically contribute to intratumoural sVEGFR1 levels and control VEGF bioavailability in a HIF-1α-dependent manner.

Next, we compared the mRNA expression of *sVEGFR1* and total *VEGFR1* in flow-sorted endothelial cells and NK cells from naive spleens from both genotypes. In the spleen, *sVEGFR1* expression in NK cells was generally lower than in endothelial cells (Fig. 3c), without genotype-specific differences in splenic NK cells from HIF-1α KO and WT mice (Fig. 3c). This might be due to the fact that the spleen is relatively well oxygenated under steady state conditions (pO_2_ = 15–25 mm Hg) compared to tumours. Interestingly, flow-sorted, tumour-associated NK cells from MC38 tumour-bearing HIF-1α KO mice showed similar expression of *sVEGFR1* at the mRNA and protein level across genotypes (Fig. 3d and Supplementary Fig. 3d). This indicates that, in contrast to splenic NK cells sVEGFR1 production in tumour-infiltrating NK cells is induced by salvage pathways and does not solely rely on *HIF-1α* expression. Therefore, the changes in vascular phenotype under these conditions are related to a reduced NK cell infiltration (Supplementary Fig. 2c). Noteworthy, the magnitude of *sVEGFR1* expression in tumour-derived NK cells was similar to tumour-sorted endothelial cells, a known source of sVEGFR1 in hypoxic tumours^4^ (Fig. 3d). This data led us to conclude that NK cells are a relevant source of intratumoural sVEGFR1, and that differential NK cell positioning within hypoxic vs. normoxic tumour regions regulates tumour angiogenesis (Supplementary Fig. 2d).

### Reconstitution of sVEGFR1 rescues the HIF-1α KO phenotype

**Fig. 4:**
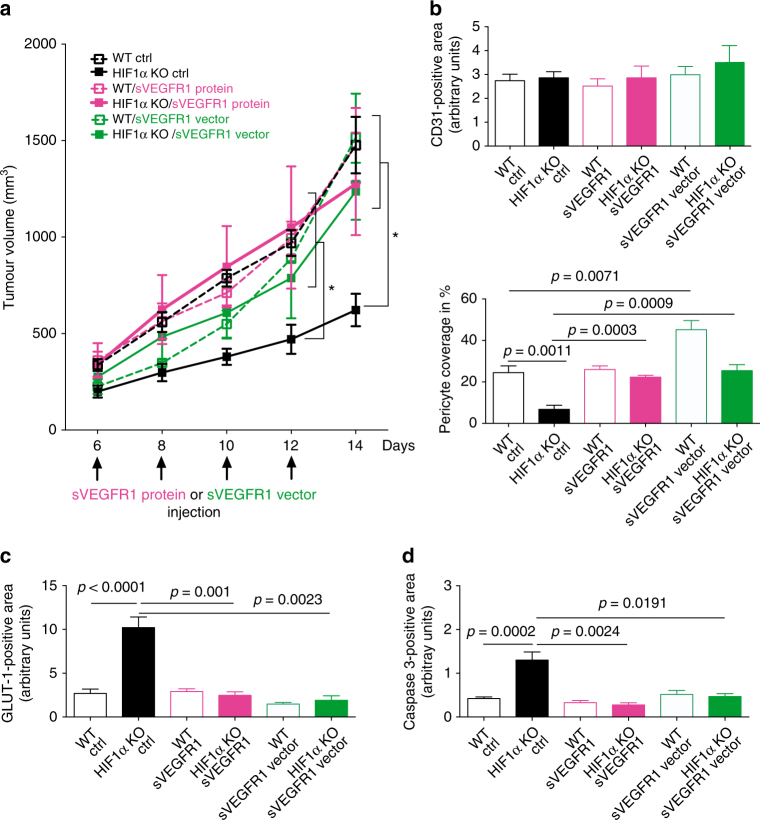
Reconstitution of sVEGFR1 rescues the HIF-1α KO phenotype. **a** Tumour volume analysis of MC38 isografts implanted in WT and HIF-1α KO mice after intratumoural injection with recombinant sVEGFR1 protein or sVEGFR1 vector at day 4, 6, 8, and 12 at endpoint, day 14. Control mice received intratumoural injections of 100 μl PBS or ctrl vector. **b** Quantitative analysis of CD31-positive endothelial cells and pericyte coverage as assessed by α-SMA/CD31 co-localisation at endpoint, day 14. **c** Quantitative analysis of hypoxic tumour areas with the specific marker GLUT1 at endpoint, day 14. **d** Quantitative analysis of caspase-3-positive areas at endpoint, day 14 (*n* = 10 for ctrl group; *n* = 5 for sVEGFR1 protein injection group; *n* = 3 for sFLT1 vector injection group). Statistical significance was determined by an unpaired Student’s *t*-test or one-way analysis of variance, where appropriate. Bars represent mean values; error bars indicate the s.e.m. Statistical significance is indicated as *P < 0.05, **P < 0.01, and ***P < 0.001

To further corroborate the role of the HIF-1-sVEGFR1 axis in NK cells for vascular remodelling and tumour growth, we next analysed the effect of sVEGFR1 reconstitution on tumour growth. For this purpose, we continuously delivered MC38-bearing WT and HIF-1α KO mice with sVEGFR1 by means of intratumoural injection of the recombinant protein. Alternatively, we employed a sVEGFR1 encoding plasmid on day 6 (when tumour growth kinetics start to differ between genotypes, see Figs. 2a, 4a), 8, 10, and 12 post tumour inoculation (Fig. 4a and Supplementary Fig. 4a). In tumour bearing WT mice, sVEGFR1 supplementation increased pericyte coverage of tumour blood vessels, reminiscent of vascular normalisation^24^ (Fig. 4b). Yet, this change in vascular morphology did not impact on tumour oxygenation (Fig. 4c), tumour cell death (Fig. 4d) or overall tumour growth (Fig. 4a). In contrast, in HIF-1α KO mice, delivery of recombinant sVEGFR1 rescued growth of MC38 tumours (Fig. 4a), along with an increase in pericyte coverage (Fig. 4b) and tumour oxygenation (Fig. 4c) as well as decrease in tumour cell death (Fig. 4d). Noteworthy, sVEGFR1 supplementation failed to rescue the infiltration defect of HIF-1α KO NK cells and did not alter the infiltration of other immune cell subsets (Supplementary Fig. 4b). These data links reduced tumour volumes to low intratumoural sVEGFR1 and non-productive angiogenesis.

### NK cell depletion recapitulates the HIF-1α KO phenotype

**Fig. 5:**
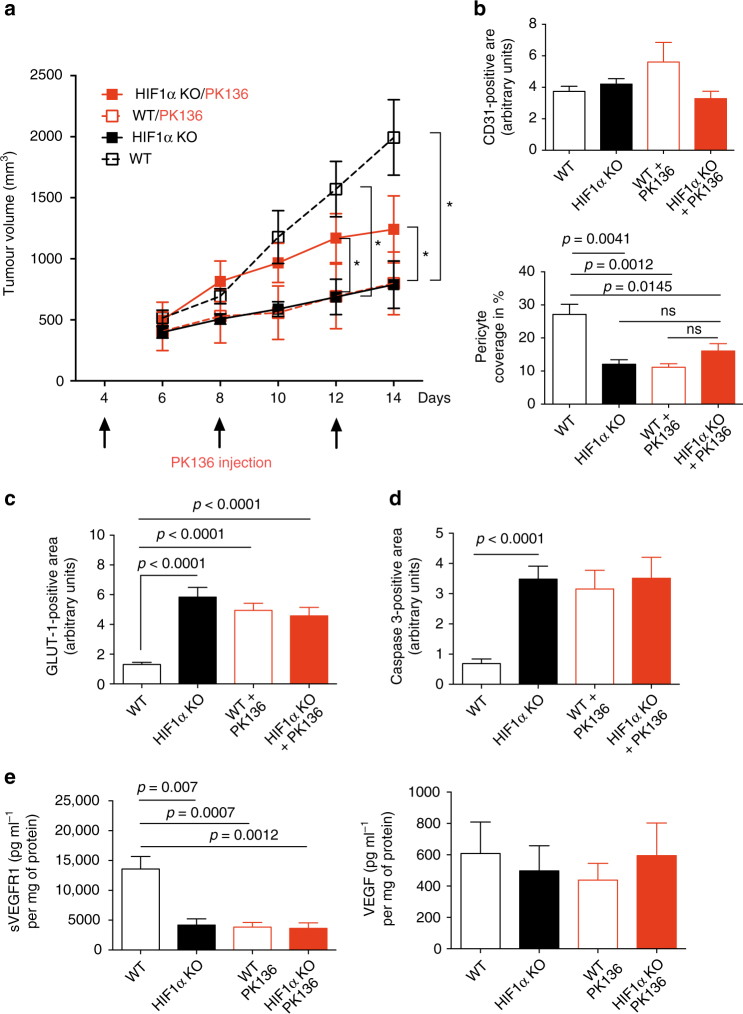
NK cell depletion recapitulates the HIF-1α KO phenotype. **a** Tumour volume analysis of MC38 isografts implanted in WT and HIF-1α KO mice injected i.p. with anti-NK1.1 monoclonal antibody PK136 (4 mg per kg body weight) at day 4, 8, and 12. Control mice received i.p. injections of 100 μl PBS. **b** Quantitative analysis of CD31-positive endothelial cells and pericyte coverage as assessed by α-SMA/CD31 co-localisation at endpoint, day 14. **c** Quantitative analysis of hypoxic tumour areas with the specific marker GLUT1 at endpoint, day 14. **d** Quantitative analysis of caspase-3-positive areas at endpoint, day 14. **e** Determination of levels of sVEGFR1 and VEGF protein in MC38 isografts implanted in WT and HIF-1α KO mice by ELISA at endpoint, day 14 (*n* = 9 for WT group; *n* = 5 for HIF-1α KO group; *n* = 8 for PK136 depleted WT group; *n* = 7 for PK136 depleted HIF-1α KO group). Statistical significance was determined by an unpaired Student’s *t*-test or one-way analysis of variance, where appropriate. Bars represent mean values; error bars indicate the s.e.m. Statistical significance is indicated as *P < 0.05, **P < 0.01, and ***P < 0.001

To study the extent of the impact of NK cells for tumour angiogenesis we depleted NK cells in MC38 tumour-bearing WT and HIF-1α KO mice^21,25^ (Supplementary Fig. 5a) on day 4, 8, and 12 (Fig. 5a). This schedule allows to avoid interference with early tumour rejection events and to achieve NK depletion in established, macroscopic tumours where vascular changes increasingly impact on growth kinetics (Figs. 2a, 5a). NK cell depletion in tumour-bearing WT mice resulted in significantly reduced tumour volumes (Fig. 5a), along with pericyte loss (Fig. 5b), increases in tumour hypoxia (Fig. 5c) and cell death (Fig. 5d), but failed to impact on other immune cell subsets (Supplementary Fig. 5b). These changes in NK cell-depleted WT tumours, reminiscent of non-functional angiogenesis, were associated with a drop in sVEGFR1 levels, whereas VEGF levels remained unchanged (Fig. 5e).

In contrast, NK cell depletion in tumour-bearing HIF-1α KO mice led to a discrete rescue of tumour growth, without impact on the vascular phenotype (Fig. 5b), tumour hypoxia (Fig. 5c), or tumour cell death (Fig. 5d). NK cell depletion was associated with an increase in CD8 T cell numbers and a decrease in F4/80 macrophages (Supplementary Fig. 5b), but these changes are unlikely to explain increased tumour volumes in NK cell-depleted HIF-1α KO mice (Fig. 5a).

These results show that NK cell depletion in established MC38 carcinomas largely phenocopies the vascular changes induced by NK cell-specific HIF-1α loss. This further suggests that NK cells can slow down angiogenesis in a sVEGFR1-dependent manner, particularly in tumours that are hardly susceptible to NK cell-mediated killing.

### NK cell HIF-1α deficiency facilitates metastasis

**Fig. 6:**
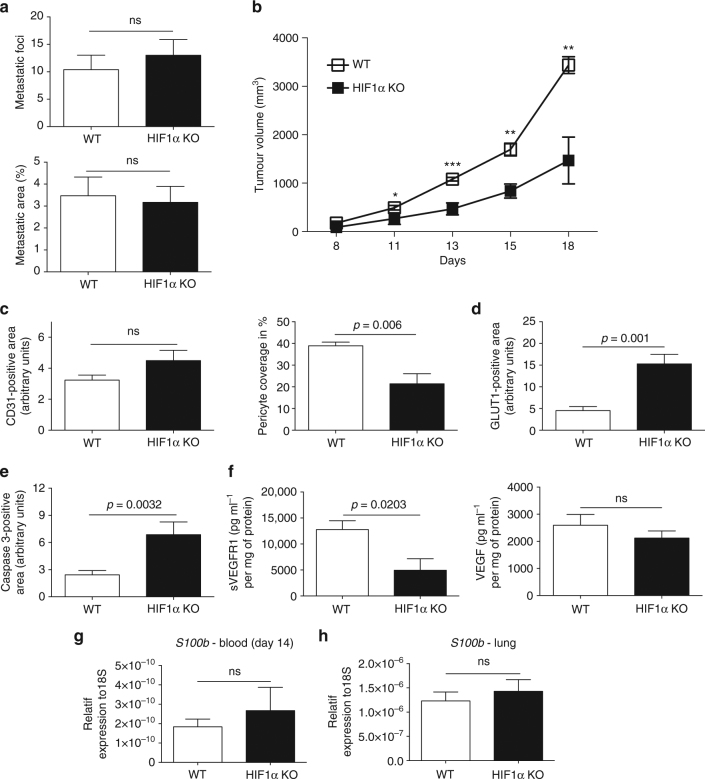
NK cell HIF-1α deficiency facilitates tumour cell intravasation and pulmonary metastasis. **a** The quantification of the metastatic foci and metastatic area in LLC tumour-bearing mice determined by H&E staining on 10 μm lung paraffin serial sections at day 14 post tumour injection. **b** Tumour volume analysis of B16F10 melanoma isografts implanted in WT and HIF-1α KO mice. **c** Quantitative analysis of CD31-positive endothelial cells and pericyte coverage as assessed by α-SMA/CD31 co-localisation at endpoint, day 14. **d** Quantitative analysis of hypoxic tumour areas with the specific marker GLUT1 at endpoint, day 14. **e** Quantitative analysis of caspase-3-positive areas at endpoint, day 14. **f** Determination of levels of sVEGFR1 and VEGF protein in B16F10 melanoma isografts implanted in WT and HIF-1α KO mice by ELISA at endpoint, day 18. **g** Gene expression analysis of the melanoma-specific gene *S100B* in peripheral blood (day 14 post tumour inoculation) and **h** lungs from melanoma-bearing animals (at endpoint, day 18 post tumour inoculation) (*n* = 10 for WT group; *n* = 6 for HIF-1α KO group). Statistical significance was determined by an unpaired Student’s *t*-test or one-way analysis of variance, where appropriate. Bars represent mean values; error bars indicate the s.e.m. Statistical significance is indicated as *P < 0.05, **P < 0.01, and ***P < 0.001

Vascular integrity is crucial to prevent metastatic spread^3,4^. We reasoned that the immature tumour blood vessel phenotype in HIF-1α KO mice may enhance tumour cell intravasation and metastatic spread. To test this idea, we analysed the lungs from WT and HIF-1α KO mice with subcutaneous LLC isografts for the pulmonary metastasis^26^. As shown in Fig. 6a, the number metastic nodules in the lungs of LLC-bearing animals was similar across genotypes, despite a pronounced reduction in the size of primary tumours from HIF-1α KO mice (Supplementary Fig. 2b). This indicates that loss of HIF1α in NK cells increases the metastatic index, which defines the relation between metastatic burden relative to tumour volume.

To further substantiate this finding, we applied an additional model of subcutaneous melanoma formation that gives rise to pulmonary metastasis^26^. Again, subcutaneous B16F10 melanomas in HIF-1α KO mice had significantly lower tumour volumes at day 18 (Fig. 6b), without genotype-specific differences in infiltration of immune cell subsets or NK cell activation (Supplementary Fig. 6a). This once more indicates that impaired tumour growth in HIF-1α KO mice does not primarily rely on NK cell cytotoxicity. Consistent with our results, loss of HIF-1α in NK cells resulted in a decrease of pericyte coverage (Fig. 6c), along with increased hypoxia (Fig. 6d) and tumour cell death (Fig. 6e). Again, we found a decrease in angiostatic sVEGFR1 levels in tumours from HIF-1α KO mice, whereas VEGF expression was similar across genotypes (Fig. 6f).

To determine the degree of melanoma cell intravasation and circulating melanoma cells as well as pulmonary metastasis, we first analysed the expression of the melanoma-specific gene *S100B* in peripheral blood (day 14 post tumour inoculation) and lungs from melanoma-bearing animals^26^ (at endpoint, day 18 post tumour inoculation). Despite the differences in tumour size at day 14 (Fig. 6b), the expression of *S100B* in blood samples from HIF-1α KO, indicative of the number of circulating melanoma cells was found elevated comparable to WT mice (Fig. 6g). In line, at endpoint (day 18) the expression of *S100B* in lungs from melanoma-bearing animals was similar across genotypes (Fig. 6h) despite pronounced differences in the primary tumour size (Fig. 6b). This verifies that HIF-1α deficiency in NK cells enhances the metastatic index.

### NK cell HIF-1α depletion promotes the VEGF null tumour growth

**Fig. 7:**
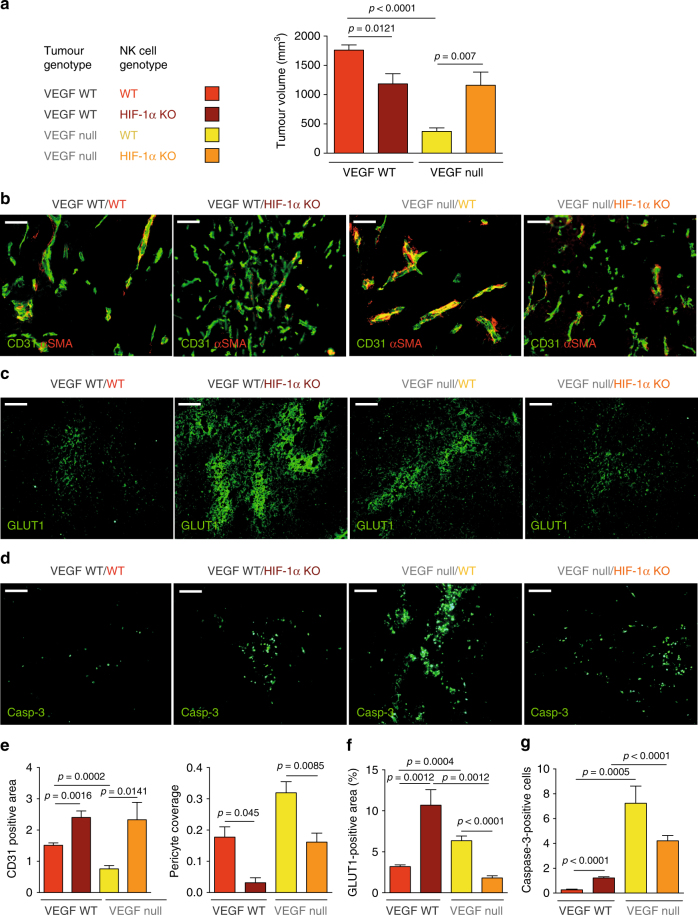
Deletion of HIF-1α in NK cells rescues the growth of VEGF-deficient tumours. **a** Tumour volume analysis of VEGF WT and VEGF null fibrosarcoma isografts implanted in WT and HIF-1α KO mice at endpoint, day 10. **b** Representative images of CD31/α-SMA immunostaining on fibrosarcoma isografts. **c** Representative images of hypoxic areas (GLUT1) in fibrosarcoma isografts. **d** Detection of apoptotic cells in fibrosarcomas by caspase-3 staining. **e** Quantitative analysis of CD31-positive endothelial cells and pericyte coverage as assessed by α-SMA/CD31 co-localisation in fibrosacromas at endpoint, day 10. **f** Quantitative analysis of hypoxic tumour areas at endpoint, day 10. **g** Quantitative analysis of caspase-3-positive areas at endpoint, day 10 (*n* = 7 for each group). Statistical significance was determined by an unpaired Student’s *t*-test or one-way analysis of variance, where appropriate. Bars represent mean values; error bars indicate the s.e.m. Statistical significance is indicated as *P < 0.05, **P < 0.01, and ***P < 0.001. Scale bar, 100 μm

Next, we sought to determine the impact of the HIF-1α-sVEGFR1-axis in NK cells on angiogenesis and growth of tumours with different levels of VEGF bioavilability. To this end, we injected isogenic VEGF-deficient fibrosarcoma cells (VEGF null)^6^, representing a model with low VEGF bioavailability (Supplementary Fig. 7a) within the tumour and the matching VEGF-expressing WT fibrosarcomas^6^ (high VEGF bioavailability) (Supplementary Fig. 7a) subcutaneously into WT and HIF-1α KO mice (Fig. 7a). Again, tumours that expressed VEGF grew significantly smaller in HIF-1α KO mice (Fig. 7a) showed lower levels of sVEGFR1 (Supplementary Fig. 7a) and enhanced VEGFR2 signalling (Supplementary Fig. 7b), resulting in non-productive angiogenesis, characterised by pericyte loss (Fig. 7b, e), increased hypoxia (Fig. 7c, f) and tumour cell death (Fig. 7d, g). Consistent with previous findings, fibrosarcomas lacking VEGF expression^6,27^ (Supplementary Fig. 7a) grew more slowly than those expressing VEGF when implanted into WT mice (Fig. 7a), due to insufficient angiogenesis (Fig. 7b, e), severe hypoxia (Fig. 7c, f), and apoptosis (Fig. 7d, g). Strikingly, however, deletion of HIF-1α in NK cells and a reduction in sVEGFR1 levels (Supplementary Fig. 7a) in VEGF null tumours rescued VEGFR2 signalling (Supplementary Fig. 7b) and tumour growth (Fig. 7a) along with restored angiogenesis (Fig. 7e) and alleviated hypoxia (Fig. 7f). This demonstrates a unique and unexpected role for HIF-1α in NK cells in the regulation of VEGF bioavailability in the tumour microenvironment and the coupling of vascular remodelling and tumour growth (summarised in Supplementary Fig. 8).

## Discussion

Like other tumour infiltrating immune cell subsets, NK cells have been shown to contribute to tumour angiogenesis by deleting individual angiogenic factors in response to various stimuli^21^. However, NK cell responses during adaptation to hypoxia, a condition that NK cells face in the tumour microenvironment, had not been addressed. By dissecting this aspect experimentally, we show that deletion of HIF-1α results in NK cell hyporeactivity upon stimulation in both, normoxia and hypoxia. Although the precise role of HIF transcription factors in NK cell function has previously not been investigated, earlier reports on the impact of hypoxia on NK cell function are inconclusive. Sarkar et al.^20^ demonstrated that hypoxia (0–1% O_2_ for 14–16 h) impaired cytotoxic action of human NK cells that can be rescued by IL-2 stimulation, whereas Velásquez et al.^28^ report a synergistic effect of short-term hypoxia (1% O_2_ for 28 h) with IL-15 (for the last 6 h)-induced NK cell activation. This highlights the importance of particular experimental conditions, e.g., degree and duration of hypoxic incubation, as well the nature of stimulatory signal, e.g., target cell type and addition of co-stimulatory cytokines. In line with this, we observe modest hypoxic suppression (2% O_2_ for 6 h) of NK cell degranulation but not IFN-γ release upon exposure to YAC-1 target cells that cannot be rescued by IL-2 or IL-15 (Fig. 1b). In contrast, hypoxia had no effect on NK cell degranulation or IFN-γ release upon stimulation with V-abl target cells.

Furthermore, we demonstrate that NK cells are a critical source of sVEGFR1, thereby negatively regulating VEGF bioavailability in the tumour microenvironment (graphical summary in Supplementary Fig. 8). Flow-sorted, tumour-associated NK cells from HIF-1α KO mice did not show a reduction of sVEGFR1 expression. It has been shown in other cell types that sVEGFR1 is co-regulated by HIF-1α and HIF-2α^4^. Therefore, in tumour-infiltrating NK cells, HIF-2α may compensate for the loss of HIF-1α during long-term exposure to hypoxia in the microenvironment. Noteworthy, the level of sVEGFR1 expression at the mRNA level in tumour-derived NK cells was as high as in tumour-sorted endothelial cells, a previously identified critical source of sVEGFR1 in hypoxic tumours^4^. Moreover, sVEGFR1 expression in NK cells was even higher than in endothelial cells when measured at the protein level by flow cytometry (Fig. 3f). These results identify NK cells as an important source of sVEGFR1 in tumours and potentially also other tissues. Given the defect in NK cell infiltration in HIF-1α KO mice, reduced sVEGFR1 levels in tumours from HIF-1α KO mice are most likely a result of a lower number of sVEGFR1-expressing NK cells (Supplementary Fig. 2c). Moreover, we show that HIF-1α KO NK cells are less present in hypoxic zones (Supplementary Fig. 2d), suggesting a failure to infiltrate or survive particularly in hypoxic tumours. Therefore, differential, HIF-1α-dependent NK cell positioning within hypoxic vs. normoxic tumour regions may contribute to the lack of NK cell-derived sVEGFR1 in hypoxic areas and subsequent non-productive angiogenesis. Interestingly, it has been shown that NK cells, rather than promote, can inhibit tumour angiogenesis upon stimulation with IL-12 in an IFN-γ-dependent manner^29^. We observe indeed reduced IFN-γ levels in HIF-1α-deficient NK cells after IL-12/18 stimulation. Therefore, low expression of angiostatic IFN-γ by NK cells in the absence of HIF-1α could contribute to non-productive angiogenesis. Moreover, in vivo reconstitution of MC38 tumours in HIF-1α KO mice with sVEGFR1 rescued the vascular phenotype and tumour growth (Fig. 4), whereas NK cell depletion of established tumours reduced sVEGFR1 levels, which led to non-productive angiogenesis and a decrease in tumour size (Fig. 5). This further substantiates the role of NK cell-derived sVEGFR1 for fine-tuning the process of vascular remodelling. In this context, it is important to mention that the vast majority NK cell depletion studies apply a protocol where NK cell depletion precedes the onset of NK cell-susceptible tumours in order to study early tumour cell rejection events; a protocol that usually results in increased tumour incidence/growth^30,31^. We applied a different protocol and achieved NK depletion in established, macroscopic tumours (4 days after inoculation of 1 × 10^7^ “less-susceptible” MC38 cells), where vascular changes increasingly impact on growth kinetics (Figs. 2a, 5a) to avoid interference with early tumour rejection events. In this setting, NK cell depletion in WT mice led to non-productive angiogenesis and a decrease in intratumoural sVEGFR1 levels along with reduced tumour growth (Fig. 5). Importantly, depletion of HIF-1α KO NK cells rescued tumour growth without inducing changes in sVEGFR1 and vascular morphology (Fig. 5). This was associated with an increase in CD8 T cells and impaired macrophage recruitment (Supplementary Fig. 5). Yet, such alterations of the immune infiltrate are believed to rather slow down tumour growth, and therefore are unlikely to contribute to tumour growth promotion in NK cell-depleted tumours from HIF-1α KO. However, we cannot exclude additional alterations in the microenvironment upon sudden removal of HIF-1α-deficient NK cells, particularly changes in the cytokine milieu and the functionality of other immune cell subsets, that may promote tumour growth.

Immature tumour vessels with decreased pericyte coverage increased permeability facilitate tumour cell intravasation and distant metastasis^3^. We observe that drastically smaller tumours from HIF-1α KO mice give rise to a degree of circulating melanoma cells and pulmonary seeds that is comparable to WT tumours (Fig. 6). This observation supports the notion that the immature vascular phenotype in tumours from HIF-1α KO mice facilitates tumour cell intravasation and distant metastasis. Moreover, the reduced cytotoxic potential in HIF-1α-deficient NK cells could impair the interception and elimination of intravasated tumour cells in the circulation, and hence facilitate pulmonary metastasis by an additional mechanism.

NK cells are known to participate in angiogenesis and vascular remodelling in the pregnant uterus^9^. Yet, in addition to vessel neoformation, vascular remodelling during pregnancy also involves maturation and even pruning of blood vessels. Therefore, the mechanism we demonstrate here could contribute to coordinated expansion of the uterine vasculature at later stages of pregnancy. However, during normal pregnancy the oxygen tension (pO_2_) in the uterus does not decrease to levels that are found in tumours (≤10 mm Hg)^22^, suggesting that HIF-1α-dependent sVEGFR1 release by NK cell is more important in the tumour setting. Consistently, female HIF-1α KO mice reproduce normally and litter sizes are similar across genotypes (data not shown).

Given the emerging importance of adoptive NK cell transfer in clinical routine, these novel findings provide a rationale to consider and target the hypoxic response in NK cells.

## Methods

### Mouse models

Experiments were conducted according to the European Community for experimental animal use guidelines (L358-86/609EEC) and were approved by the Ethical Committee of INSERM. Targeted deletion of HIF-1α in NKp46-expressing NK cells was achieved by crossing the loxP-flanked HIF-1α allele^14^ to the *Ncr1* (NKp46) promoter-driven cre recombinase^15^ (termed HIF-1α KO mice). To mitigate the influence of strain variation, mice were kept in a >99% C57Bl/6J background. Adult HIF-1α KO mice did not show obvious phenotypical changes, e.g., size, body weight, susceptibility to infections; life span. To generate isografts, 1 × 10^6^ V-abl lymphoma cells, 1 × 10^6^ B16F10 melanoma cells, 1 × 10^7^ LLC cells or MC38 colon carcinoma cells on a BL6 background (ATCC) were injected subcutaneously into Ncr1Cre-/HIF-1α^+f^/^+f^ (WT) and Ncr1Cre + /HIF-1α^+f^/^+f^ mice in a volume of 100 μl PBS. Mouse embryonic fibroblasts (MEFs) were isolated from mice both alleles of exon 3 of VEGF-A flanked by loxP sites (VEGF^+f^/^+f^ mice)^6^. The transgenic MEFs were immortalised by stable transfection with SV40 large T antigen and then transformed with a vector expressing oncogenic mutant H-Ras (Val-12). Subsequently, the VEGF^+f^/^+f^ MEFs were infected with an adenovirus expressing Cre recombinase to delete exon 3 of the *VEGF* gene^6^. A total of 5 × 10^6^ of VEGF null or WT MEF’s were injected subcutaneously into mice in a volume of 100 μl PBS. Data are expressed as mean ± SEM. Statistical significance was determined by ANOVA or unpaired *t*-test.

### Depletion of NK cells

Randomised cohorts of the tumour-bearing WT and HIF-1α KO mice mice were injected i.p. with anti-NK1.1 monoclonal antibody PK136 (4 mg per kg body weight; kindly provided by Prof. Veronika Sexl from Vienna) at day 4, 8, and 12. Control mice received i.p. injections of 100 μl PBS.

### sVEGFR1 injection

Randomised cohorts of the tumour-bearing WT and HIF-1α KO mice received intratumoural injections of 250 ng of recombinant, active, carrier-free murine sVEGFR1 (R&D Systems, 7756-FL-050) reconstituted in PBS every 2 days starting on day 6 until endpoint at day 14. Control mice were injected with PBS.

### sVEGFR1-expressing plasmid

Aortic arch was excised from C57BL/6J mice. Total RNA was extracted from aortic arch by using the RNeasy mini kit (Qiagen K.K, Tokyo, Japan) and reversely transcribed with SuperScript III First Strand Synthesis System (Thermo Fisher Scientific K. K., Yokohama, Japan). Murine sFlt-1 (D88690.1) was amplified by polymerase chain reaction (PCR) using KOD DNA polymerase (Toyobo, Osaka, Japan) and the primers: sense 5′-CCCAAGCTTATGGTCAGCTGCTGGGACACC-3′ and antisense 5′-AAAGCGGCCGCGAGACAACTGTTACTTTTCAAATGAGTCCT-3′. PCR products were digested with HindIII and NotI, and subcloned into pcDNA3 expression vector (Thermo Fisher Scientific K.K.).

### Preparation of PEI–DNA complexes and injections

For in vivo administration, FLT1 and ctrl plasmid DNA was complexed with in vivo-jetPEI (Polyplus Transfection, Illkirch, France), according to the manufacturer’s guidelines. 15 μg of DNA per one injection was complexed with in vivo-JetPEI at an N/P ratio of 6 in 5% glucose solution for intratumoural injection. The mixture was incubated for at least 30 min at room temperature in order for the complexes to form before being injected into the mice. Randomised cohorts of the tumour-bearing WT and HIF-1α KO mice received intratumoural injections of 15 μg of DNA in 100 μl of 5% glucose solution every 2 days starting on day 6 until endpoint at day 14. Control mice were injected with control plasmid.

### Flow cytometry

Single-cell suspension of BM, spleen, liver, and tumour were obtained and stained. Intracellular stainings for Granzyme B and IFN-γ were performed using Cytofix/Cytoperm (BD-Bioscience). Cell viability was measured using LIVE/DEAD® Fixable Aqua Dead Cell Stain Kit (Thermo Fisher). Flow cytometry was carried out on a FACS LSR II (Becton-Dickinson). Data were analysed using FlowJo (Treestar). The following mAbs from eBioscience or BD-Biosciences or BioLegend were used: anti-CD19 (1D3; 562291), anti-CD3 (145-2C11; 562286), anti-CD4 (GK1.5; BLE100408), anti-CD8 (53-6.7; BLE100723), anti-F4/80 (BM8; BLE123118), anti-CD11c (N418; BLE117310), anti-MHC II (M5/114.15.2; 11-5321-85), anti-NK1.1 (PK136; 12-5941-82), anti NKp46 (29A1.4; 25-3351-82), anti-CD11b (M1/70; 560455), anti-CD27 (LG.7F9; 12-0271-81), anti-CD45 (30-F11; 45-0451-82), anti-Ly49H (3D10; 13-5886-82), anti-Ly49D (4e5; 13-5782-82), anti-Ly49C/I (5E6; 557418), anti-NKG2AB6 (16a11; 12-5897-83), anti-NKG2D (CX5; 12-5882-81), anti-KLRG1 (2F1; 561620), anti-CD43 (S7; 560663), anti-CD49a (HMα1; 142604), anti-CD49b (DX5; 47-5971-80), anti-Granzyme B (NGZB; 12-8898-82), anti-IFN-γ (XMG1; 554413), anti-CD107a (1D4B; 553793), and relevant isotype controls.

For VEGFR1 detection following antibodies were used: for visualise total VEGFR1 (anti-VEGFR1-Alexa Fluor 488; Abcam; ab195253; dilution 1/50) and for membrane-anchored VEGFR1 (rabbit anti-VEGFR1; Abcam; ab2350; dilution 1/50; with secondary antibody goat anti-rabbit IgG AF647; dilution 1/500). The gating strategy is depicted in Supplementary Fig. 9.

### Splenocyte isolation and stimulation

Splenic lymphocytes were prepared and cultured in the presence of GolgiStop (BD) without or with cytokines (rmIL-15 100 ng ml^−1^; rmIL-12 25 ng ml^−1^; rmIL-18 5 ng ml^−1^; rhIL-2 1000 U ml^−1^), or on antibody-coated plates (anti-NK1.1 at 1-3-10 μg ml^−1^) or with PMA (20 ng ml^−1^) and ionomycin (1 μg ml^−1^) or with one of the following tumoural cell lines MC38, LLC, or v-ABL (400,000 cells per well) for 6 h at 37 °C in hypoxia/normoxia culturing conditions in the presence of anti-CD107a (1D4B; 560648). Surface and intracellular stainings for granzyme B (NGZB; 12-8898-82) and IFN-γ (XMG1; 554413) were performed using Cytofix/Cytoperm (BD-Bioscience). Cell viability was measured using LIVE/DEAD^®^ Fixable Aqua Dead Cell Stain Kit (Thermo Fisher).

### NK cell purification

NK cells were purified using NK Cell Isolation Kit II (Miltenyi) an LS Column (Miltenyi), and a MidiMACS™ Separator (Miltenyi).

### In vitro cytotoxicity assays

In vitro cytotoxicity assays were performed with purified, splenic, naive NK cells and the MC38, LLC and V-abl tumour cell lines. Target cells were washed and labelled with CFSE (Sigma-Aldrich). Following the washing steps, NK cells were co-cultured with target cells at E:T ratios of 1:1 and 10:1 for 6 h at 37 °C and 5% CO_2_ in RPMI 1640 medium (Invitrogen) supplemented with 10% fetal bovine serum (FBS), 50 U/ml penicillin-streptomycin (Lonza) in normoxia (20% of O_2_) and hypoxia (2% of O_2_). Culture of target cells alone was used as a negative control. Each experimental condition was performed in three replicates. Then cells were washed with PBS containing 0.5% BSA (Miltenyi Biotec) and labelled with a Live/Dead Fixable Aqua Dead Cell Stain Kit (Invitrogen), according to the manufacturer’s protocol before data acquisition on a BD LSRII flow cytometer (BD Biosciences, PARCC, Paris). The data were analysed using FlowJo (Treestar). The target cells were identified as CFSE+, and effector cells were identified as CFSE−. The dead target cells were identified as CFSE+Live/Dead+. Spontaneous death was defined as the proportion of dead target cells cultured alone (negative control), and this value was subtracted from the proportion of dead target cells cocultured with effector cells. Each cytotoxicity assay was repeated in at least three independent experiments.

### In vivo fluorescence imaging of angiogenesis and hypoxia

Fluorescence acquisition were performed using the planar optical camera PhotonImager RT (Biospace Lab, France). Mice were narcotised with isoflurane (2–3% isoflurane, 0.5 l per minute air) and injected intravenously with 10 nmol of AngioStamp^®^700 (50 μmol L^−1^ in PBS; Fluoptics, France) or with 2 nmol of HypoxiSense 680 (20 µmol L^−1^ in PBS; Perkin Elmer, USA) for imaging angiogenesis (expression of αvβ integrin, overexpressed in neovessel endothelial cells during angiogenesis) or hypoxia (expression of carbonic anhydrase 9 protein, which increases in hypoxic regions within tumours), respectively. Images were acquired before and 5 min, 1, 2, 4, 6, 12, 24, 48 post tracer injection. Filters were set as following: excitation filter: 650 nm, emission filter: 575 nm, background filter: 575 nm. Images analyses was performed using the software M3Vision (Biospace Lab, France). Grayscale photographic images and fluorescence colour images were superimposed. Regions of interest were drawn over each tumour to determine the signal intensity.

### Histology, immunohistochemistry, and immunofluorescence

After removal, tumours and lungs were fixed in 4% paraformaldehyde and then embedded in paraffin. 7 μm sections were deparaffinised with xylene and rehydrated with graded ethanol. The sections were stained according to routine immunohistochemistry procedures and visualised by Vectastain ABC or ABC-AP kit (Vector Laboratories, Burlingame, CA). Alternatively, samples were frozen in OCT and fixed in acetone/methanol before standard immunofluorescence procedures.

Primary antibodies used for immmunohistochemistry and immunofluorescence: Rat anti-CD31 at 1:200 dilution (BD Pharmingen; 550274), rat anti-CD31 (DIANOVA; DIA-310), biotinylated mouse anti-SMA-alpha at 1:200 (Thermo Scientific; 14-9760-82), rat anti-NKp46 (BioLegend; 137606), rabbit anti-GLUT-1 (Abcam; ab652) at 1:500, rabbit anti-cleaved caspase-3 (Cell Signalling; 9661) at 1:500. The fluorochrome-conjugated Alexa 488 (A11070; A11006; A11017) and Alexa 568 (A11077; A11031) were used as secondary antibodies (1:200).

Lung metastases in the LLC model was analysed by Haematoxylin and Eosin (H&E) staining on 10 μm lung paraffin serial sections at day 14 post tumour injection^26^.

### Quantitative analysis of histologic markers

For quantitative analysis of the distribution of immunohistochemical markers within the tumour, the midline sections of tumours were photographed into TIFF images using a ZEISS Axioskope 2 plus microscope and ZEISS Axiocam camera system and the area (number of pixels) with positive staining equal to or greater than a set threshold was measured using the ImageJ programme and such marked areas were normalised by the number of images for each tumour. To determine vessel density, the vasculature marked by CD31 was skeletonised using the ImageJ programme and the area covered by blood vessels was determined. To determine pericyte coverage of blood vessels, CD31/α-SMA colocalisation was quantified.

### Immunoprecipitation and immunoblotting

Tumours were lysed in RIPA buffer and 500 μg of lysate were used for immunoprecipitation of VEGFR2. The following antibodies were used in this study: rabbit anti-VEGFR2 (55B11, Cell Signalling; 2479), HRP-conjugated anti-phosphotyrosine (4G10^®^, Millipore; 16-105), goat anti-Albumin (Abcam; ab19194), and HRP-conjugated mouse anti-β-actin (Santa Cruz; sc-47778). For a quantitative analysis the membranes were scanned with a fluorescence scanner and the signal strength was determined by using ImageQuant^®^ software. Full-size images are presented in Supplementary Fig. 10.

### ELISA

Concentrations of VEGF-A and sVEGFR1 in tumours and aliquots of supernatants were determined using commercial kits (Quantikine ELISA Immunoassay, R&D Systems) and expressed in pg ml^−1^ per mg of whole tissue protein.

### Gene expression by quantitative PCR

*General procedure*: Cells were lysed and tissue samples were homogenised in RLT buffer (Quiagen). Total RNA was isolated with Quiagen RNA extraction kits following the manufacturer’s instructions. For real-time PCR analysis, the isolated RNA was reverse transcribed (Eurogentec). For PCR reactions, TaqMan Mastermix (Eurogentec) was used. 50 ng cDNA was used as template to determine the relative amount of mRNA by real-time PCR (BioRad Detection System). PCR conditions were as follows: 95 °C for 10 min followed by 40 cycles of 95 °C for 15 s and 60 °C for 1 min. Data were normalised to 16S mRNA levels. The following primers were used: 16s forward primer: 5′-AGATGATCGAGCCGCGC-3′, reverse primer: 5′-GCTACCAGGGCCTTTGAGATGGA-3′; VEGF-A forward primer: 5′-ATCCGCATGATCTGCATGG-3′, reverse primer: 5′-ATCCGCATGATCTGCATGG-3′; soluble VEGFR1 forward primer: 5′-GTCACAGATGTGCCGAATGG-3′, reverse primer: 5′-TGACTTTGTGTGGTACAATC-3′; PDGF-beta forward primer: 5′-CATCCGCTCCTTTGATGATCTT-3′, reverse primer: 5′-ATGAGCTTTCCAACTCGACTCC-3′; FGF-2 forward primer: 5′-GGCTTCTTCCTGCGCATCCA-3′, reverse primer: 5′-GCTCTTAGCAGACATTGGAAGA-3′; PlGF forward primer: 5′-TGCTGGTCATGAAGCTGTTC-3′, reverse primer: 5′-GGACACAGGACGGACTGAAT-3′; Angiopoietin-1 forward primer: 5′-GATCTTACACGGTGCCGATT-3′, reverse primer: 5′-TTAGATTGGAAGGGCCACAG-3′; Angiopoietin-2 forward primer: 5′-TCCAAGAGCTCGGTTGCTAT-3′, reverse primer: 5′-AGTTGGGGAAGGTCAGTGTG-3′; FLT-1 (extracellular domain): forward primer: 5′-CATTGTAAACGTGAAACCTCAGATCT-3′, reverse primer: 5′-CTGCTGCCCAGCGGATAG-3′; FLT-1 (intracellular domain): forward primer: 5′-GGGAAAGGAGTCCTGCTGTTCT-3′, reverse primer: 5′-GAGCGGAATAGGTGTAAACTCATAGAT-3′.

The expression of the melanocyte specific gene *S100b* was measured as readout of lung colonisation by qRT-PCR following reverse transcription to cDNA with the QuantiTect Reverse Transcription kit and custom made primers for S100b and 18S (Qiagen).

Gene expression analysis on purified NK cells and endothelial cells from tumours and spleens: 4500 NK cells and endothelial cells were directly lysed and analysed according to the CellsDirect™ protocol (CellsDirect™ One-Step qRT-PCR Kits, Invitrogen). A low abundance targets, *sFLT1* and total *FLT1*, were detected using a TaqMan^®^ probe. The following TaqMan^®^ probes were used: FLT-1 (extracellular domain): 5′-FAM-CCGTGTCCTCGCTTCCAAGCCC-TAMRA-3′; FLT-1 (intracellular domain): probe 5′-FAM-ACCCCCAGACTACAACTCCGTGGTGT-TAMRA-3′. Data were normalised to 18S (commercial Taqman probe: Mm03928990_g1).

### Cell culture

Cell lines (LLC; MC38, colon carcinoma; YAC-1, Mus musculus lymphoma; B16F10, Mus musculus skin melanoma) were obtained from ATCC and V-abl cell line kindly provided by Prof. Veronika Sexl from Vienna. Tumour cells were cultured in Dulbecco’s Modified Eagle’s Medium (DMEM High Glucose), supplemented with 10% FBS, 2 mM glutamine, 50 U ml^−1^ penicillin and 100 μg ml^−1^ streptomycin at 37 °C in a humidified atmosphere of 5% CO_2_ in air and were checked for Mycoplasma contamination. YAC-1, splenocytes, and isolated NK cells were maintained in RPMI supplemented with 10% FBS. Murine endothelial cells were grown in endothelial cell growth medium (Lonza).

### Statistical analysis

Statistical analysis was performed with the Prism 6.0 software (GraphPad Software). Statistical significance was determined by an unpaired Student’s *t*-test or one-way analysis of variance, where appropriate. Statistical significance is indicated as *P < 0.05, **P < 0.01, ***P < 0.001, and ****P < 0.0001.

### Data availability

The data that support the findings of this study are available from the corresponding author upon request.

## Electronic supplementary material

Supplementary Information
