# Supplementary material for: Loss of HIF-1α in natural killer cells inhibits tumour growth by stimulating non-productive angiogenesis
Source: Nat Commun. 2017 Nov 17;8:1597. doi: 10.1038/s41467-017-01599-w (PMC5694012; doi:10.1038/s41467-017-01599-w)
Supplement: Supplementary file 1 — Supplementary Information [file 41467_2017_1599_MOESM1_ESM.pdf]

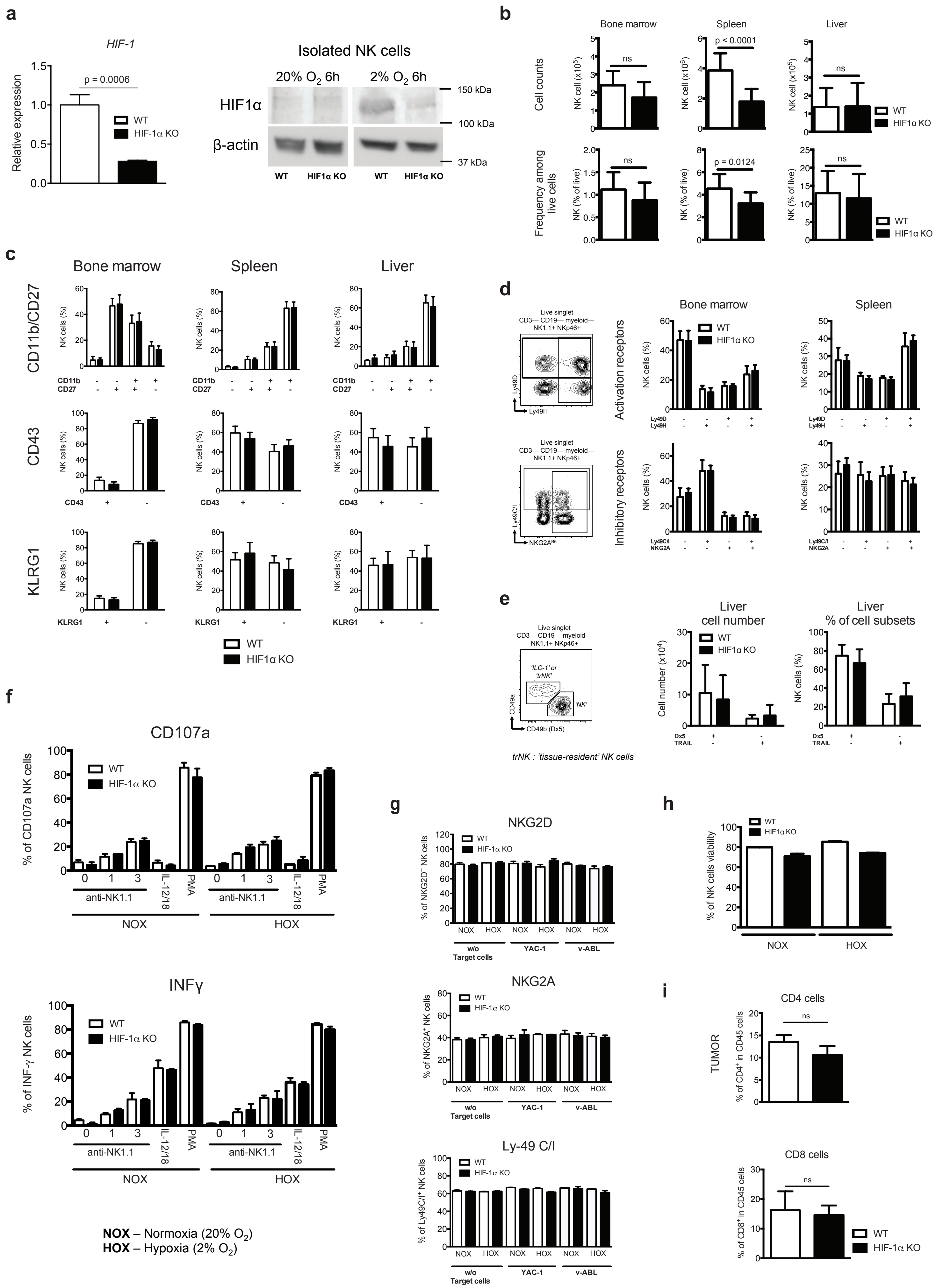
a
HIF-1
Relative expression
p = 0.0006
WT
HIF-1α KO
Isolated NK cells
20% O2 6h
2% O2 6h
HIF1α
β-actin
150 kDa
100 kDa
37 kDa
WT
HIF1α KO
b
Bone marrow
Spleen
Liver
Cell counts
Frequency among live cells
ns
p < 0.0001
p = 0.0124
WT
HIF1α KO
c
Bone marrow
Spleen
Liver
CD11b/CD27
CD43
KLRG1
NK cells (%)
CD11b
CD27
WT
HIF1α KO
d
Live singlet
CD3— CD19— myeloid—
NK1.1+ NKp46+
Ly49D
Ly49H
Ly49C/I
NKG2A
Activation receptors
Inhibitory receptors
Bone marrow
Spleen
e
CD49a
CD49b (Dx5)
'ILC-1' or 'trNK'
'NK'
Liver cell number
Liver % of cell subsets
Cell number (x10^4)
Dx5
TRAIL
trNK : 'tissue-resident' NK cells
f
CD107a
% of CD107a NK cells
WT
HIF-1α KO
anti-NK1.1
IL-12/18
PMA
NOX
HOX
INFγ
% of INF-γ NK cells
NOX – Normoxia (20% O2)
HOX – Hypoxia (2% O2)
g
NKG2D
% of NKG2D+ NK cells
NKG2A
% of NKG2A+ NK cells
Ly-49 C/I
% of Ly49C/I+ NK cells
w/o Target cells
YAC-1
v-ABL
h
% of NK cells viability
i
CD4 cells
CD8 cells
TUMOR
% of CD4+ in CD45 cells
% of CD8+ in CD45 cells

Supplementary Figure 1. (a) HIF-1α expression analysis (left) and HIF-1α protein level analysis (right) in isolated (splenic) NK cells from WT (n=5) and HIF-1α KO (n=5) mice after 6h of incubation in normoxia (20% of O2) and hypoxia (2% of O2). (b) The number and the frequency of NK cells in lymphocytes from WT (n=11) and HIF-1α KO (n=12) mice in different tissues (bone marrow, spleen, liver). (c) The expression analysis of CD27, CD11b, CD43 and KLRG1 on gated NK cells from WT (n=11) and HIF-1α KO (n=12) mice in different tissues (bone marrow, spleen, liver). (d) The expression analysis of the NK cell activating and inhibitory receptors Ly49D, Ly49H, Ly49C/I and NKG2AB6 on gated NK cells from WT (n=11) and HIF-1α KO (n=12) mice in different tissues (bone marrow, spleen). (e) The expression analysis of CD49a and CD49b on gated NK cells from liver from WT (n=11) and HIF-1α KO (n=12) mice. (f) Splenocytes from WT (n=4) and HIF-1α KO (n=3) mice were stimulated with ligand mediated activation of the NK1.1 receptor (1-3µg ml-1), activating cytokines IL-12/18 (20ng ml-1) or unspecific activation by PMA (200ng ml-1) /Ionomycin (1µg ml-1) under normoxia (NOX – 20% of O2) and hypoxia (HOX – 2% of O2) for 6 hours in vitro. NK cell degranulation (CD107A+) and IFN-γ expression were analysed by flow cytometry. (g) The expression analysis of NK cell activating and inhibitory receptors NKG2D, NKG2A and Ly-49 C/I in WT (n=5) and HIF-1α KO (n=5) mice. (h) The NK cell viability in WT (n=5) and HIF-1α KO (n=5) mice. (i) The LLC tumour infiltrating lymphocytes analysis from WT (n=3) and HIF-1α KO (n=3) mice showing percentages of CD4-, CD8- positive cells among CD45 cells at endpoint. Bars represent mean values; error bars indicate the s.e.m. Statistical significance was determined by an unpaired Student’s t-test or one-way analysis of variance, where appropriate. Statistical significance is indicated as *$P<0.05$, **$P<0.01$ and ***$P<0.001$.

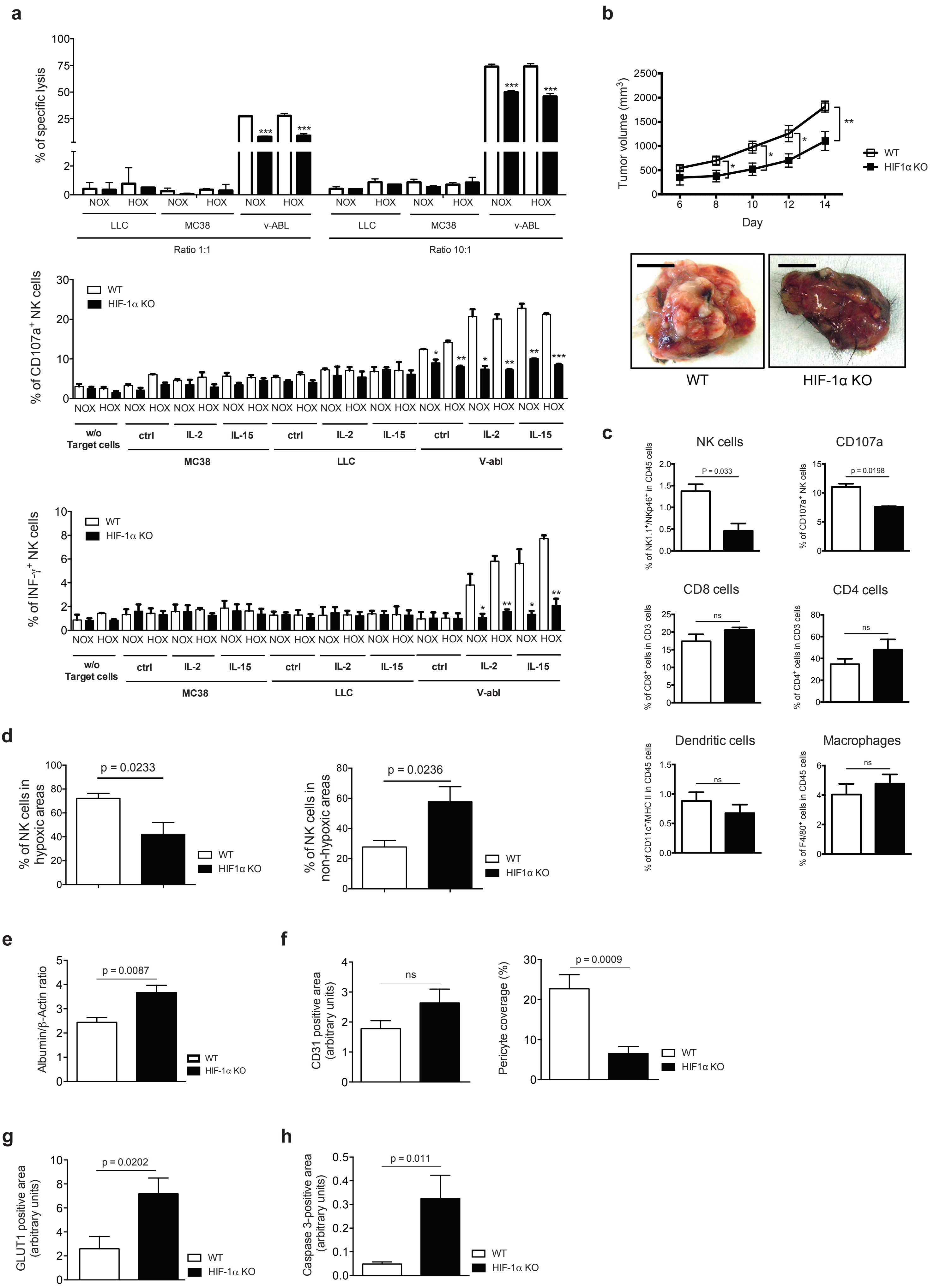

a
% of specific lysis
100
75
50
25
2
0
NOX HOX NOX HOX NOX HOX NOX HOX NOX HOX NOX HOX
LLC MC38 v-ABL LLC MC38 v-ABL
Ratio 1:1
Ratio 10:1
***
% of CD107a+ NK cells
WT
HIF-1α KO
w/o Target cells
ctrl IL-2 IL-15 ctrl IL-2 IL-15 ctrl IL-2 IL-15
MC38
LLC
V-abl
% of INF-γ+ NK cells
b
Tumor volume (mm3)
2500
2000
1500
1000
500
0
6 8 10 12 14
Day
WT
HIF1α KO
WT
HIF-1α KO
c
NK cells
P = 0.033
% of NK1.1+/NKp46+ in CD45 cells
CD107a
p = 0.0198
% of CD107a+ NK cells
CD8 cells
ns
% of CD8+ cells in CD3 cells
CD4 cells
ns
% of CD4+ cells in CD3 cells
Dendritic cells
ns
% of CD11c+/MHC II in CD45 cells
Macrophages
ns
% of F4/80+ cells in CD45 cells
d
p = 0.0233
% of NK cells in hypoxic areas
WT
HIF1α KO
p = 0.0236
% of NK cells in non-hypoxic areas
e
p = 0.0087
Albumin/β-Actin ratio
WT
HIF-1α KO
f
ns
CD31 positive area (arbitrary units)
p = 0.0009
Pericyte coverage (%)
WT
HIF1α KO
g
p = 0.0202
GLUT1 positive area (arbitrary units)
WT
HIF-1α KO
h
p = 0.011
Caspase 3-positive area (arbitrary units)
WT
HIF-1α KO

Supplementary Figure 2. (a) Top : Cytotoxic assay of purified NK cells from WT (n=5) and HIF-1α KO mice (n=5) with target cells (MC38, LLC and Vabl lymphoma cells, Ratios E/T 1 :1 and 10 :1) under normoxia (NOX – 20% of O2) and hypoxia (HOX – 2% of O2) for 6h. Middle and bottom: Splenocytes from WT and HIF-1α KO mice were stimulated with target cells (MC38, LLC and V-abl lymphoma cells, Ratio E/T 1:1), in absence or presence of rhIL-2 and rmIL15 under normoxia (NOX – 20% of O2) and hypoxia (HOX – 2% of O2). NK cell degranulation (CD107A+) and INF-γ expression were analysed by flow cytometry (n=4 for each group). (b) Tumour volume analysis of LLC isografts injected subcutaneously in WT and HIF-1α KO mice with representative images at endpoint, day 14 (n=12). Scale bars in macroscopic figures indicate 5 mm. (c) Flow cytometry analysis for NK1.1, NKp46 (for NK cells), CD107a (for NK cell activation state) CD3, CD4, CD8 (for T cells) CD11c, MHCII (for Dendritic cells), F4/80 (for Macrophages) on LLC isografts from WT and HIF-1α KO mice at endpoint, day 14 (n=5). (d) Quantitative analysis of NK cell localization relative to hypoxic and non-hypoxic areas within the tumour by NKp46 staining and the hypoxia-inducible surrogate marker Glut1 (n=5). (e) Quantitative analysis of intratumoural amounts of albumin for LLC tumours from WT and HIF-1α KO mice (n=5). (f) Quantitative analysis of CD31-positive endothelial cells (n=9) and pericyte coverage as assessed by α-SMA/CD31 co-localization (n=9) in LLC tumours. (g) Quantitative analysis of hypoxic tumour areas by Glut1 staining on LLC isografts (n=9). (h) Quantitative analysis of caspase-3 positive areas on LLC isografts (n=7). Statistical significance was determined by an unpaired Student's t-test or one-way analysis of variance, where appropriate. Bars represent mean values; error bars indicate the s.e.m. Statistical significance is indicated as *P<0.05, **P<0.01 and ***P<0.001.

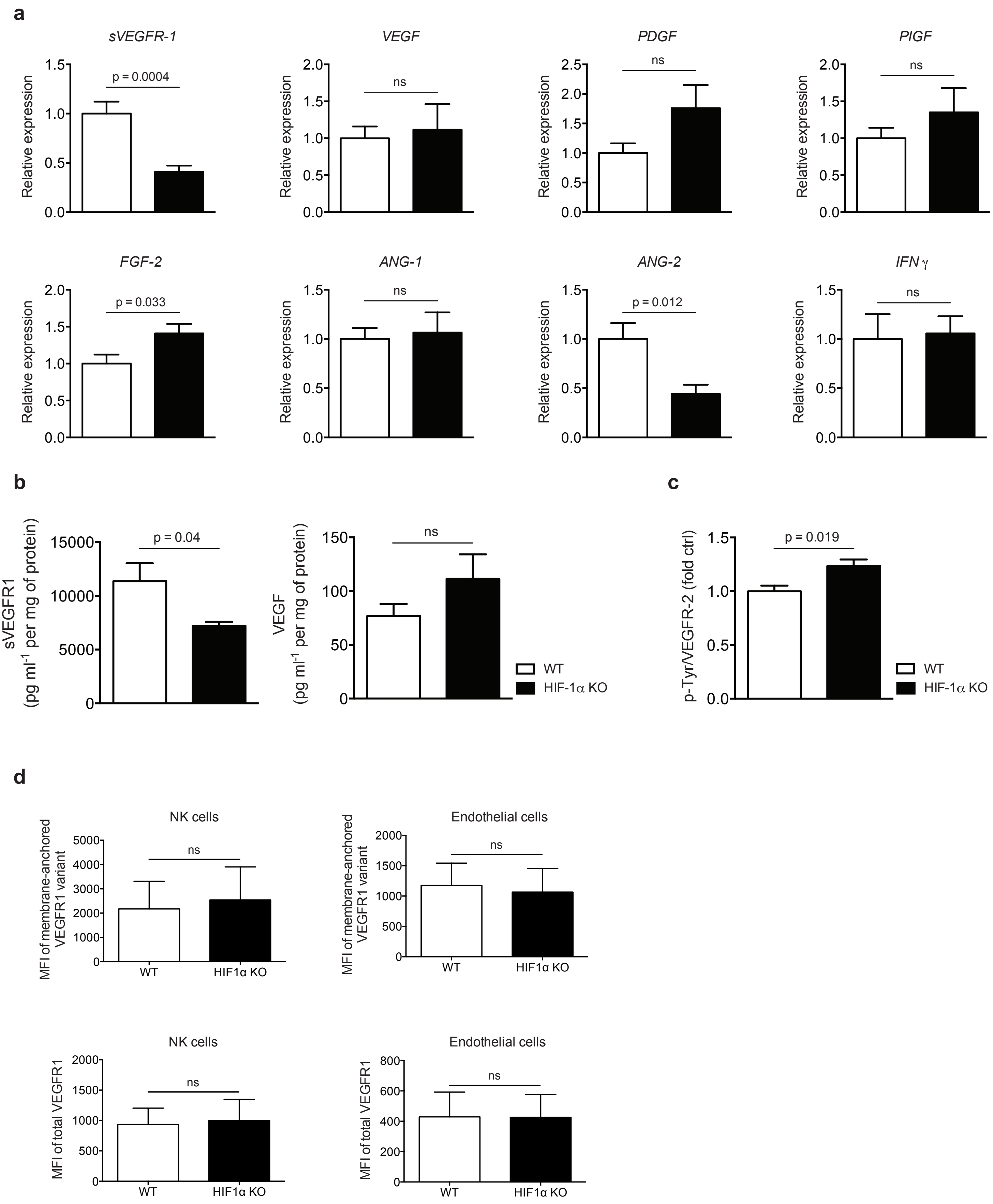
a
sVEGFR-1
p = 0.0004
VEGF
ns
PDGF
ns
PlGF
ns
FGF-2
p = 0.033
ANG-1
ns
ANG-2
p = 0.012
IFN γ
ns
Relative expression
b
sVEGFR1
(pg ml-1 per mg of protein)
p = 0.04
VEGF
(pg ml-1 per mg of protein)
ns
WT
HIF-1α KO
c
p-Tyr/VEGFR-2 (fold ctrl)
p = 0.019
WT
HIF-1α KO
d
NK cells
MFI of membrane-anchored VEGFR1 variant
ns
WT
HIF1α KO
Endothelial cells
MFI of membrane-anchored VEGFR1 variant
ns
WT
HIF1α KO
NK cells
MFI of total VEGFR1
ns
WT
HIF1α KO
Endothelial cells
MFI of total VEGFR1
ns
WT
HIF1α KO

Supplementary Figure 3. (a) Gene expression analysis for angiogenic and angiostatic factors (sVEGFR1, VEGF, PDGF, PIGF, FGF-2, ANG-1, ANG-2, INF-γ) in LLC tumours injected subcutaneously in WT and HIF-1α KO mice at endpoint, day 14 (n=10). (b) Determination of levels of sVEGFR1 and VEGF protein in LLC tumours injected subcutaneously in WT and HIF-1α KO mice by ELISA at endpoint, day 14 (n=5). (c) Ratio of p-Tyr and VEGFR2 signal intensities as a measure of receptor activation at endpoint, day 14 (n=5). (d) Flow cytometry analysis of membrane-anchored VEGFR1 and total VEGFR1 for NK cells (NK1.1+/NKp46+) and endothelial cells (CD31+/CD45-) on MC38 tumours from WT and HIF-1α KO mice at endpoint, day 10 (n=15). Bars represent mean values; error bars indicate the s.e.m.. Statistical significance was determined by an unpaired Student's t-test or one-way analysis of variance, where appropriate. Statistical significance is indicated as *P<0.05, **P<0.01 and ***P<0.001.

a

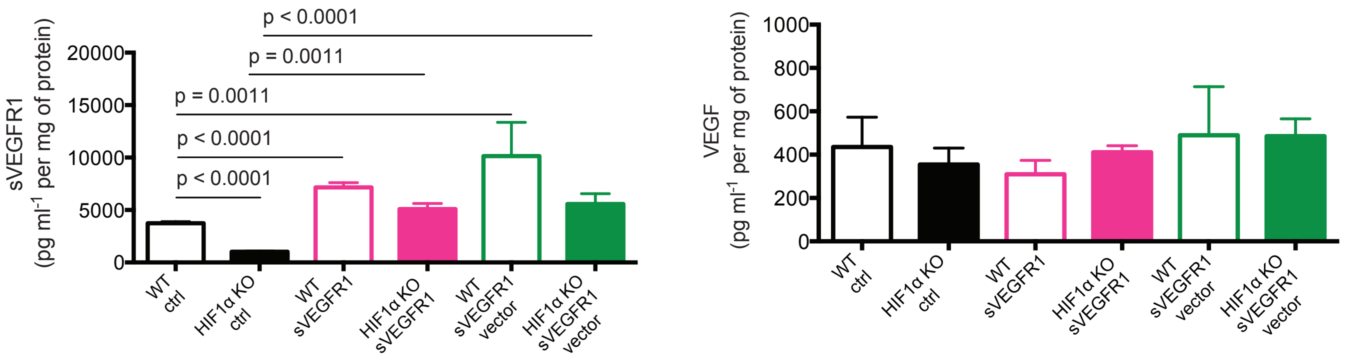

b

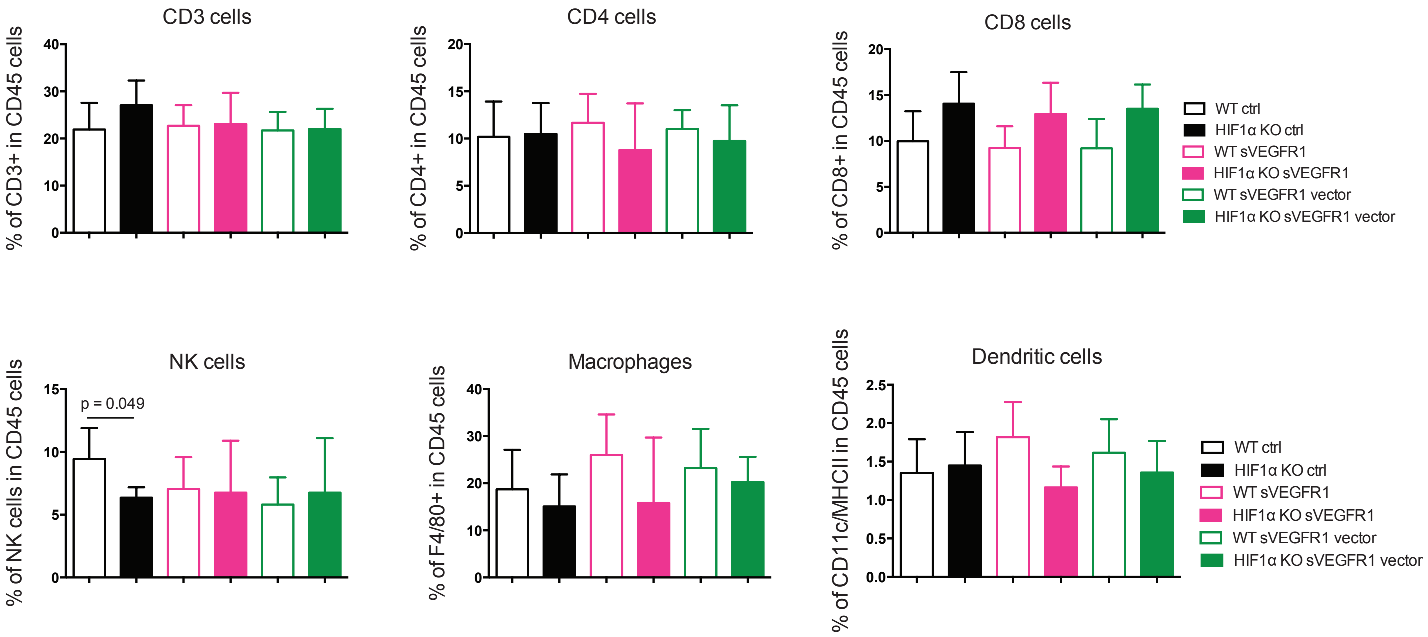

Supplementary Figure 4. (a) Determination of levels of sVEGFR1 and VEGF protein in MC38 isografts implanted in WT and HIF-1α KO mice after intratumoural injection with recombinant sVEGFR1 protein or sVEGFR1 vector at day 4, 6, 8 and 12 by ELISA at endpoint, day 14. Control mice received intratumoural injections of 100 µl PBS or ctrl vector. (b) Flow cytometry analysis for CD3, CD4, CD8 (for T cells), NK1.1, NKp46 (for NK cells), F4/80 (for Macrophages), CD11c, MHCII (for Dendritic cells) on MC38 isografts from WT and HIF-1α KO mice after intratumoral injection with recombinant sVEGFR1 protein or sVEGFR1 vector as previously described, at endpoint day 14 ; (n=10 for ctrl group ; n=5 for sVEGFR1 protein injection group ; n=3 for sFLT1 vector injection group). Student's t-test was performed to calculate P value. Bars represent mean values; error bars indicate the s.e.m. Statistical significance is indicated as *P<0.05, **P<0.01 and ***P<0.001.

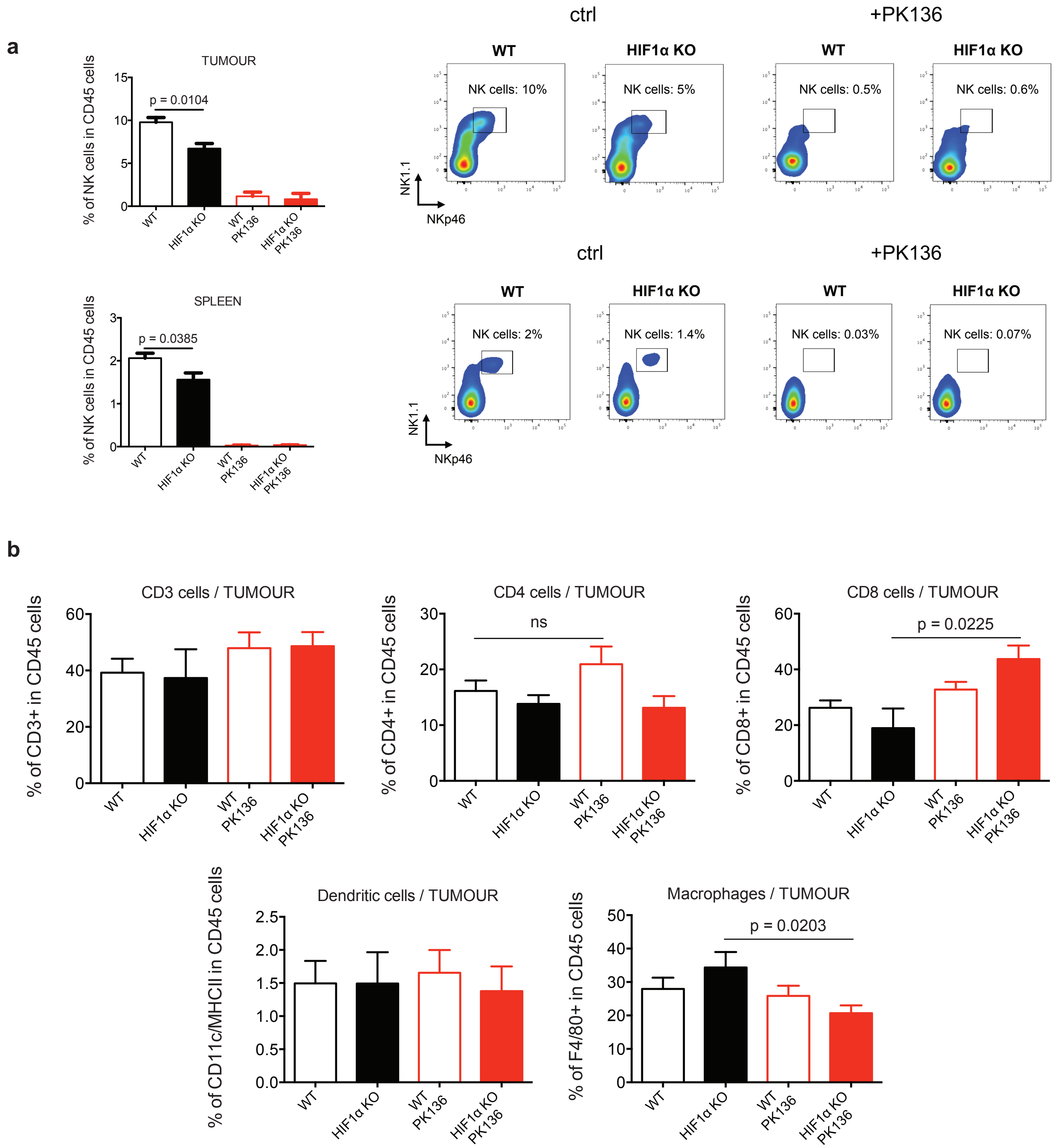

Supplementary Figure 5. (a) Flow cytometry analysis of spleen and tumour samples for NK1.1, NKp46 (for NK cells) on PK136 depleted-MC38 isografts implanted in WT and HIF-1α KO mice with representative dot-plots (b) Flow cytometry analysis for CD3, CD4, CD8 (for T cells), CD11c, MHCII (for Dendritic cells), F4/80 (for Macrophages) on MC38 isografts implanted in WT and HIF-1α KO mice treated as previously described ; (n=9 for WT group ; n=5 for HIF-1α KO group ; n=8 for PK136 depleted WT group ; n=7 for PK136 depleted HIF-1α KO group). Student's t-test was performed to calculate P value. Bars represent mean values; error bars indicate the s.e.m. Statistical significance is indicated as *P<0.05, **P<0.01 and ***P<0.001.

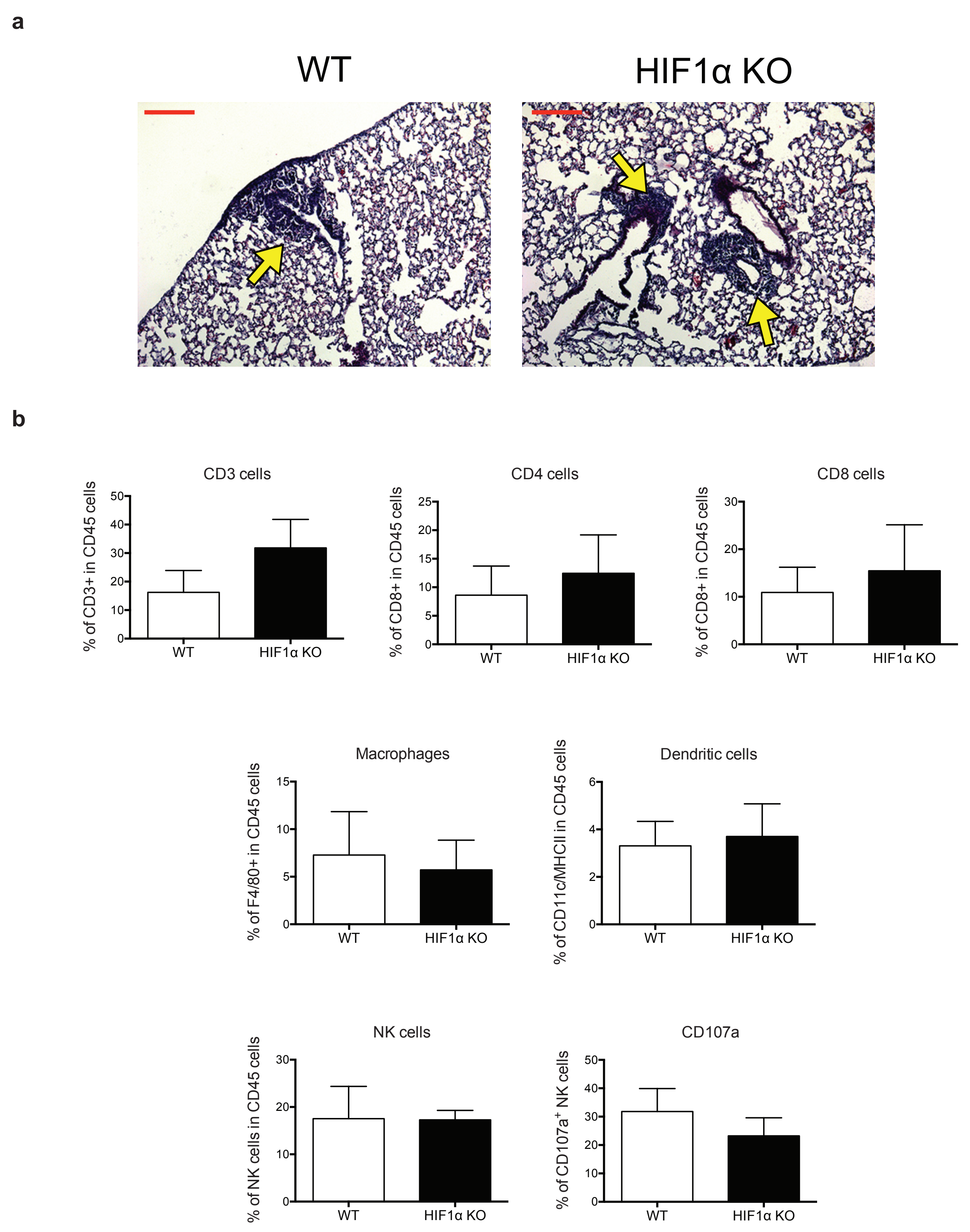

Supplementary Figure 6. (a) Representative lung H&E sections of metastases from LLC tumour–bearing wild-type (WT) and HIF-1α KO mice. Arrows point to metastatic foci. (b) Flow cytometry analysis for CD3, CD4, CD8 (for T cells), F4/80 (for Macrophages), CD11c, MHCII (for Dendritic cells), NK1.1, NKp46 (for NK cells), CD107a (for NK cell activation state) on B16F10 melanoma isografts from WT and HIF-1α KO mice at endpoint, day 18 (n=10 for WT group ; n=6 for HIF-1α KO group). Bars represent mean values; error bars indicate the s.e.m.; statistical significance was determined by an unpaired Student's t-test. Statistical significance is indicated as *P<0.05, **P<0.01 and ***P<0.001. Scale bar, 100 µm.

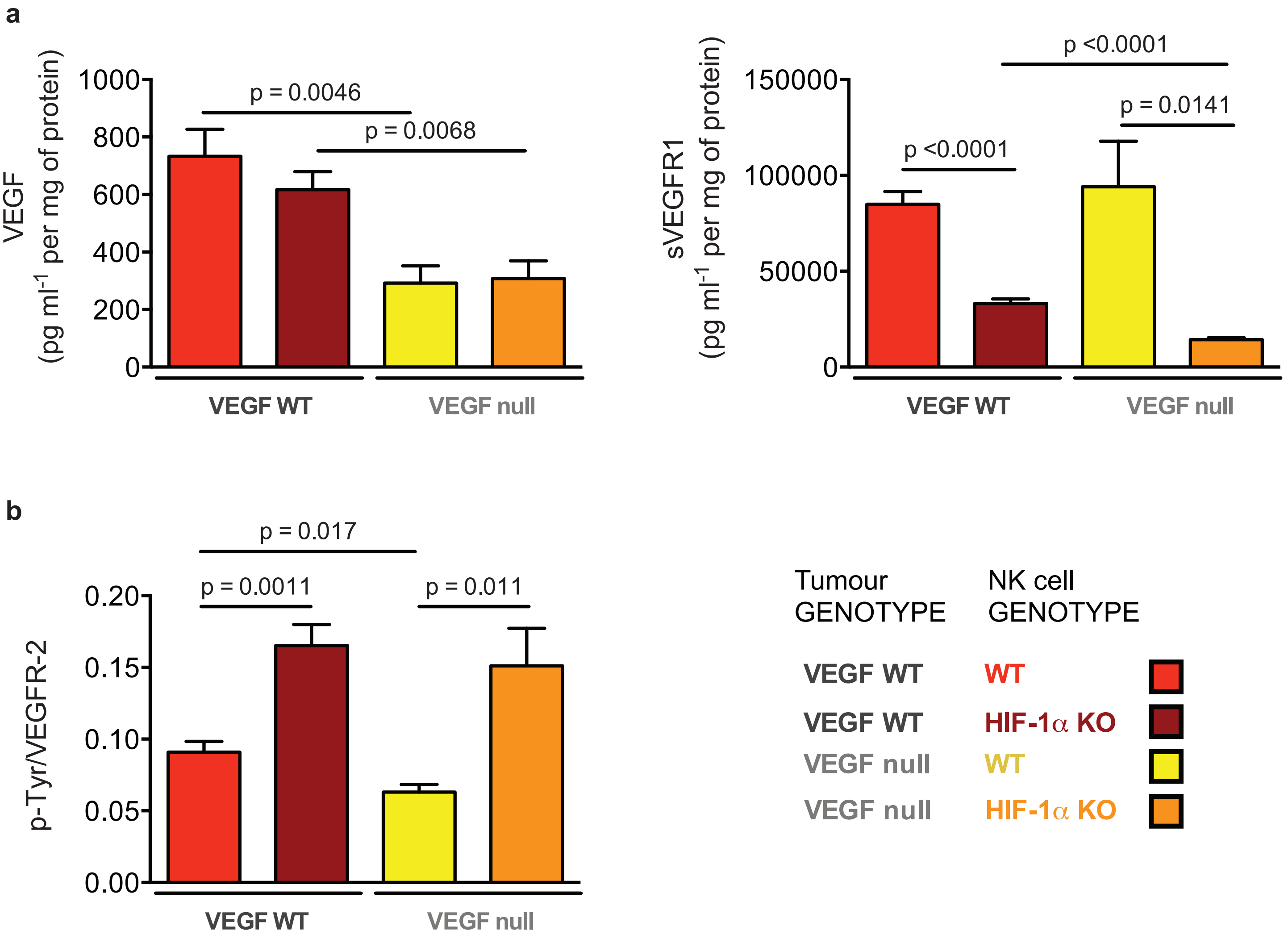

Supplementary Figure 7. (a) Determination of levels of VEGF and sVEGFR1 protein in VEGF WT and VEGF null fibrosarcoma isografts implanted in WT and HIF-1α KO mice by ELISA at endpoint, day 10 (n=6). (b) Ratio of p-Tyr and VEGFR2 signal intensities as a measure of receptor activation at endpoint, day 10 (n=6). Student's t-test was performed to calculate P value. Bars represent mean values; error bars indicate the s.e.m. Statistical significance is indicated as $*P<0.05$, $**P<0.01$ and $***P<0.001$.

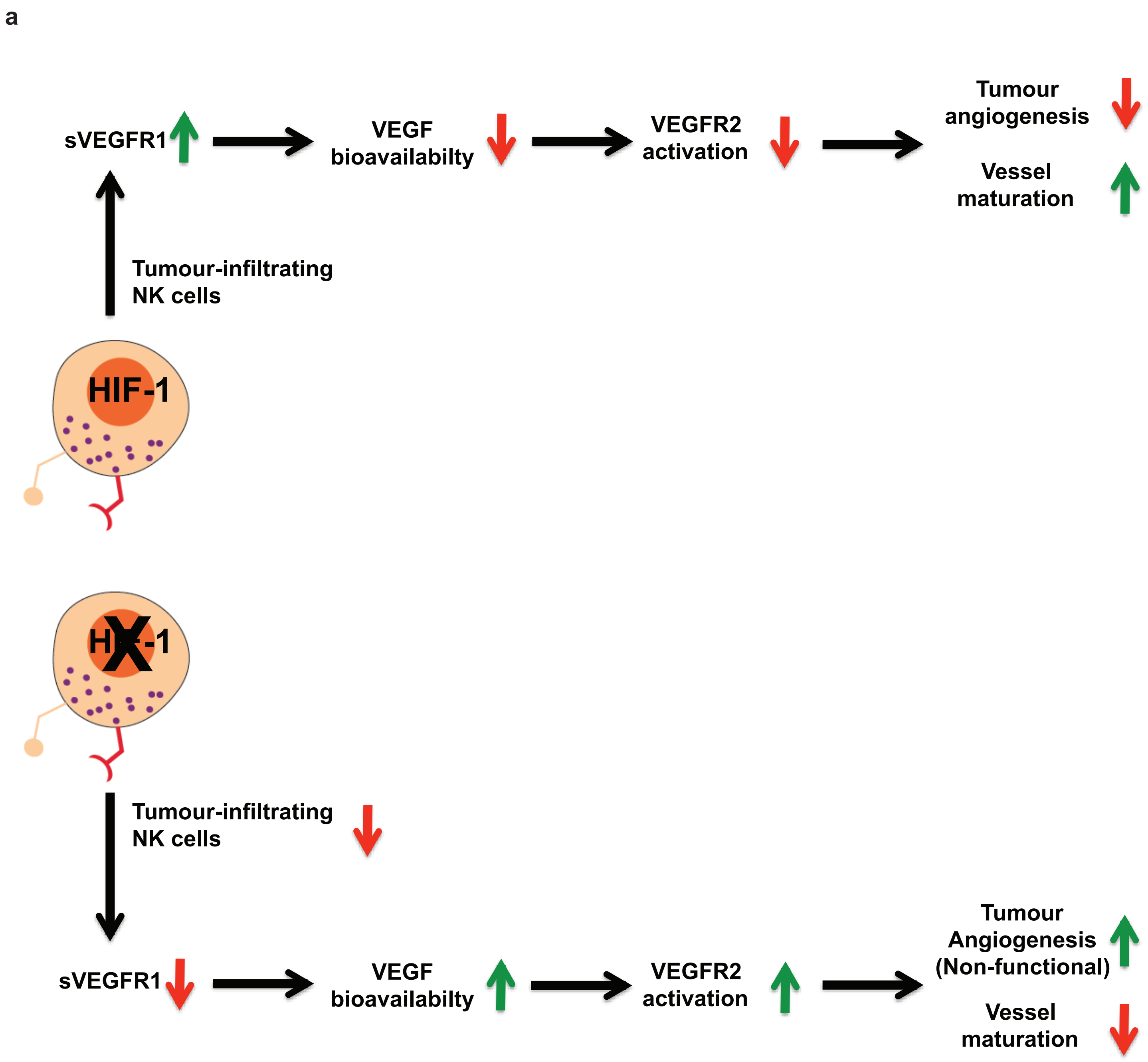

Supplementary Figure 8. (a) Graphical summary and proposed mechanism: NK cells are an important source of sVEGFR1, thereby negatively regulating VEGF bioavailability and pathological angiogenesis in a HIF-1α-dependent manner (green arrows – increase ; red arrows – decrease).

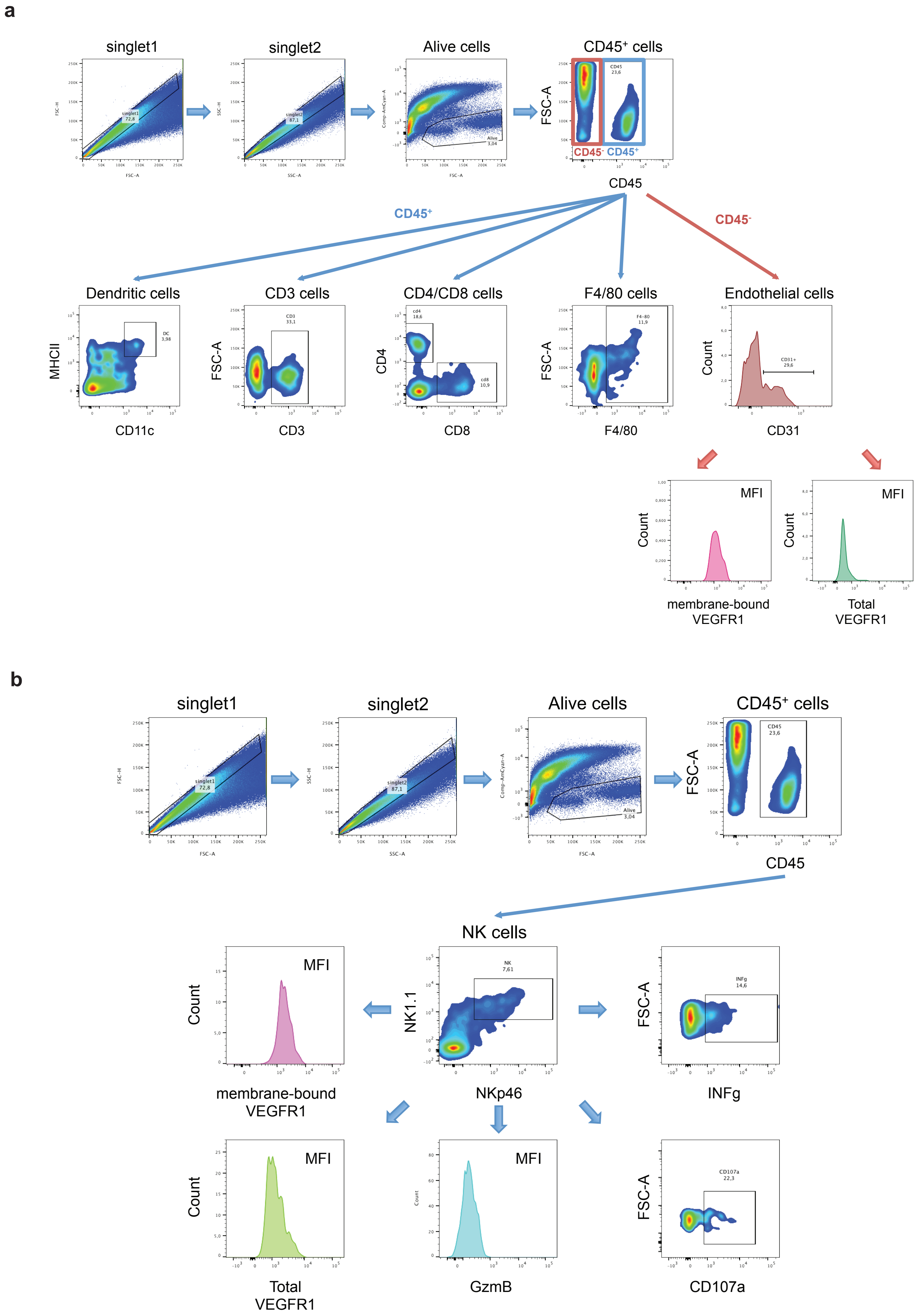
a
singlet1
singlet2
Alive cells
CD45⁺ cells
FSC-A
CD45⁻ CD45⁺
CD45
CD45⁺
CD45⁻
Dendritic cells
CD3 cells
CD4/CD8 cells
F4/80 cells
Endothelial cells
MHCII
CD11c
FSC-A
CD3
CD4
CD8
FSC-A
F4/80
Count
CD31
MFI
Count
membrane-bound VEGFR1
MFI
Count
Total VEGFR1
b
singlet1
singlet2
Alive cells
CD45⁺ cells
FSC-A
CD45
NK cells
MFI
Count
membrane-bound VEGFR1
NK1.1
NKp46
FSC-A
INFg
MFI
Count
Total VEGFR1
MFI
GzmB
FSC-A
CD107a

Supplementary Figure 9. Gating strategy: The single cell leukocyte population was selected by FSC-H versus FSC-A and SSC-H versus SSC-A. The leukocyte population was further analysed for their uptake of the Live/-Dead Aqua stain to determine live versus dead cells and for the expression of CD45. (a) Then CD45+ cells were classified as dendritic cells by co-expression of CD11c and MHCII, T cells by CD3 expression, T helper cells by CD4 expression and T cytotoxic cells by CD8 expression, macrophage population by F4/80 expression. Endothelial cells were classified as CD45- and CD31 positive cells and they were analysed for membrane-bound VEGFR1 and total VEGFR1. (b) Then CD45+ cells were classified as NK cells by co-expression of NKp46 and NK1.1 and they were analysed for membrane-bound VEGFR1, total VEGFR1, granzyme B, CD107a and interferon γ.

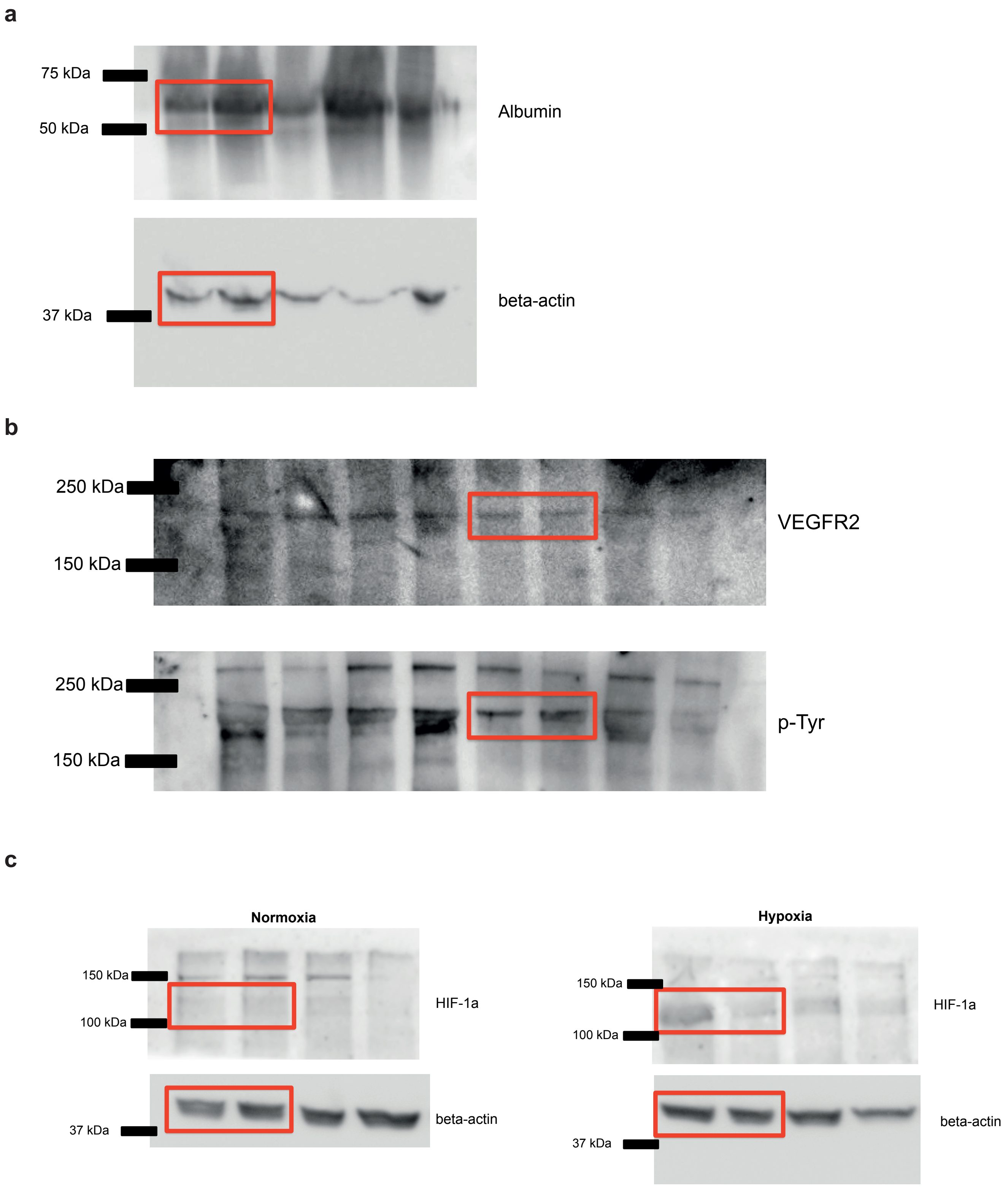

Supplementary Figure 10. Scans of Western blots shown in Figures 2c (a), 3b (b) and Supplemental Figure 1a (c). Molecular weight markers are indicated. Red boxes highlight the lanes that are displayed in the corresponding Figures.
